# Functionalization of Ti64 via Direct Laser Interference Patterning and Its Influence on Wettability and Oxygen Bubble Nucleation

**DOI:** 10.1021/acs.langmuir.3c02863

**Published:** 2024-01-31

**Authors:** Julian Heinrich, Fabian Ränke, Karin Schwarzenberger, Xuegeng Yang, Robert Baumann, Mateusz Marzec, Andrés Fabián Lasagni, Kerstin Eckert

**Affiliations:** † Institute of Fluid Dynamics, Helmholtz-Zentrum Dresden-Rossendorf, Bautzner Landstr. 400, Dresden 01328, Germany; ‡ Institute of Process Engineering and Environmental Technology, Technische Universität Dresden, Helmholtzstr. 14, 01069 Dresden, Germany; § Institute of Manufacturing Science and Engineering, Technische Universität Dresden, George-Baehr-Str. 3c, 01069 Dresden, Germany; ∥ Academic Centre for Materials and Nanotechnology, AGH University of Krakow, Av. Mickiewicza 30, 30-059 Krakow, Poland; ⊥ Fraunhofer Institute for Material and Beam Technology IWS, Winterbergstraße 28, 01277 Dresden, Germany

## Abstract

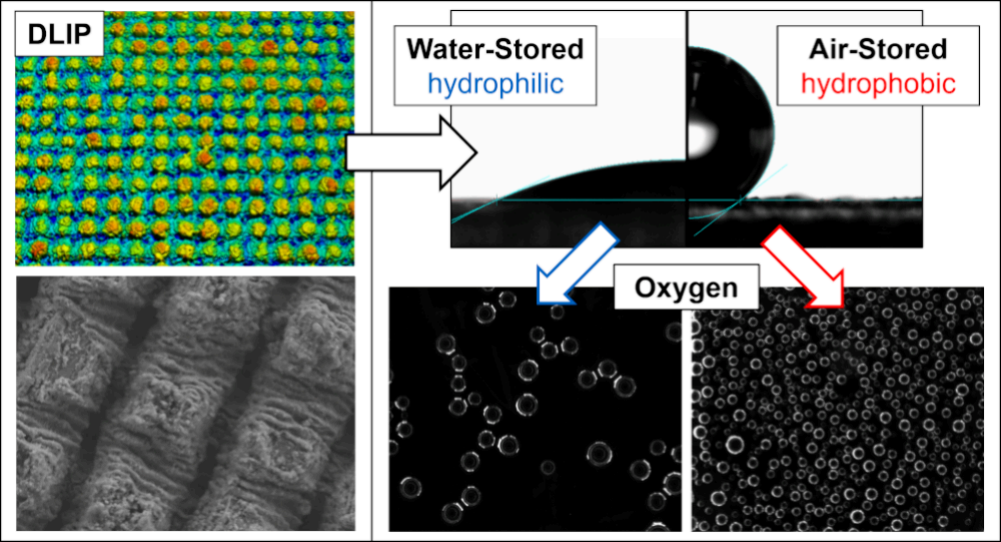

The nucleation of bubbles on solid surfaces is an important phenomenon in nature and technological processes like electrolysis. During proton-exchange membrane electrolysis, the nucleation and separation of the electrically nonconductive oxygen in the anodic cycle plays a crucial role to minimize the overpotential it causes in the system. This increases the efficiency of the process, making renewable energy sources and the “power-to-gas” strategy more viable. A promising approach is to optimize gas separation by surface functionalization in order to apply a more advantageous interface to industrial materials. In this work, the connection between the wettability and bubble nucleation of oxygen is investigated. For tailoring the wettability of Ti64 substrates, the direct laser interference patterning method is applied. A laser source with a wavelength of 1064 nm and a pulse duration of 12 ps is used to generate periodic pillar-like structures with different depths up to ∼5 μm. The resulting surface properties are characterized by water contact angle measurement, scanning electron microscopy, confocal microscopy, and X-ray photon spectroscopy. It was possible to generate structures with a water contact angle ranging from 20° up to nearly superhydrophobic conditions. The different wettabilities are validated based on X-ray photon spectroscopy and the different elemental composition of the samples. The results indicate that the surface character of the substrate adapts depending on the surrounding media and needs more time to reach a steady state for deeper structures. A custom setup is used to expose the functionalized surfaces to oxygen-oversaturated solutions. It is shown that a higher hydrophobicity of the structured surface yields a stronger interaction with the dissolved gas. This significantly enhances the oxygen nucleation up to nearly 350% by generating approximately 20 times more nucleation spots, but also smaller bubble sizes and a reduced detachment rate.

## 1 Introduction

With the growing need to replace fossil fuels, different alternatives for power generation and storage came into focus over the last years. More and more renewable energy sources based on solar power, wind power, and tidal energy are used. However, a major disadvantage of these facilities is their high fluctuation in electricity due to weather and seasonal conditions, with temporal variations strongly affecting the network stability and resilience. A high excess of electrical power could lead in extreme cases to a failure of the system due to the overload.^1,2^ On the other hand, the renewable sources also suffer from temporary low availability and the so-called dunkelflaute issue, which describes a time period in which little electrical energy can be generated due to the absence of wind or sunlight. Thus, better storage technologies are essential to bridging these periods.^3^

One auspicious possibility to increase the energy system flexibility is the “power-to-gas” strategy, which aims to use the temporal excess of green electricity from wind parks and solar power plants and feed it to electrolyzer stacks.^4^ Here, the electrical energy is converted via electrolysis into chemical energy and stored in form of hydrogen.^5,6^ H_2_ features the highest gravimetric energy density of approximately 140 MJ/kg among common fuels and emits only water during its usage without any carbon emissions.^7^ Proton-exchange membrane (PEM) electrolysis is a promising technique for H_2_ generation and features an energy conversion of 60–80% but being costly due to its need of noble metals and the high cost of components.^8−11^ To increase the attractiveness of PEM, many different approaches are currently under investigation to enhance its efficiency, one of them being enhanced oxygen separation in the anodic cycle. Since the generated gas is not electrically conductive, it increases ohmic resistance and overpotential in the porous transport layers and therefore the energy consumption of the electrolysis.^12,13^ The working principle of the current gas–liquid separators is based on buoyancy,^14^ so only the gaseous O_2_ is accessible for the separation process while the dissolved O_2_ cannot be removed. Through manipulation of the surface chemistry and morphology of the materials in contact with the process water, their hydrophilic/hydrophobic character and therefore the wettability can be changed, which will result in an enhanced or hindered transition of the O_2_ from the supersaturated liquid to the gaseous phase.

Although pure titanium itself is not catalytically active in the electrochemical process, it can be used as a substrate for electrodes, since its surface features excellent processability toward a high surface enlargement.^15,16^ Furthermore, in the PEM electrolyzer stack, it is used as material for the construction of the bipolar plates and the current collectors.^16^ One of its alloys is Ti64, which is widely used in a variety of industrial applications, like in aerospace engineering due to its low density and high mechanical stability.^17^ Additionally, it features an enhanced corrosion resistance with respect to the acidic condition in the anodic compartment of PEM electrolyzers compared to conventional metal compounds, such as stainless steel. Furthermore, it exhibits a better heat resistance and a lower flammability compared to pure titanium.^18,19^ At the same time, Ti64 can be easily processed by laser-based approaches. In particular, direct laser interference patterning (DLIP) is a laser texturing technique that has shown the capability to generate microstructured surfaces with feature sizes down to the submicrometer range, allowing the implementation of several surface functionalities.^20^ Aside from the precise and reproducible features of the laser patterning, which create very well-defined wetting properties, additional advantages of the method are a high substrate purity due to the noncontact processing and the high-energy input in the form of heat. The ablation process in the heat-affected zones and the generated microtextures lead to a very strong increase in surface area. As a result of the laser–matter interaction, the surface chemistry is also modified by thermal influence due to the high-energy laser radiation process at ambient air, which results in a hydrophilic surface oxide layer for metallic materials and an increase of free surface energy.^21−23^ In this context, Kuisat et al. demonstrated that additively manufactured Ti64 equipped with line-like DLIP features can be beneficial for water-repellent and anti-icing surface properties.^24^ DLIP in conjunction with ultrashort pulses (picosecond range) enables the creation of high-precision microtextures. This is primarily due to the ablation process involved, which results in minimal heat-affected zones and strongly reduces material remelting.^25^ By combining the DLIP with ultrashort laser pulses multiscale textures can be produced, which further improve the surface functionality as well as their mechanical durability.^26^ The aim of this study is to investigate the influence of DLIP on the wetting behavior and the nucleation of the O_2_ bubble on Ti64 substrates. This is based on the idea that O_2_ is a nonpolar gas and its dissolved solutes are attracted due to hydrophobic interactions and will form aggregates in aqueous environments to maximize the hydrogen bondings of water.^27^ These aggregates have higher attraction forces to hydrophobic surfaces due to the higher void probability, as shown by Li et al. previously.^28^ Hereby, these voids are low-density regions in the liquid caused by the disordered movement of molecules and the general density fluctuations.^29^ To gain a deeper understanding of the interaction between surface character and O_2_ bubble nucleation, the Ti64 substrates are first fabricated with highly periodic pillar-like DLIP structures with a spatial period of 6.0 μm and two different structure depths. The topography of the generated surface is analyzed via confocal microscopy, and the long-term wettability is studied via static contact angle measurements over a period of 50 days during which the substrates were in contact with the two media, either water or air, mimicking two different storage conditions. The interaction between chemical surface composition and wettability is analyzed in detail via X-ray photoelectron spectroscopy (XPS) measurements, giving a deeper insight of the aging of the DLIP structures depending on the surrounding media. Lastly, O_2_ bubble nucleation tests in oversaturated water are performed and show promising results to tune the O_2_ nucleation in industrial applications, such as PEM electrolyzer stacks.

## 2 Materials and Methods

### 2.1 Materials

**Figure 1:**
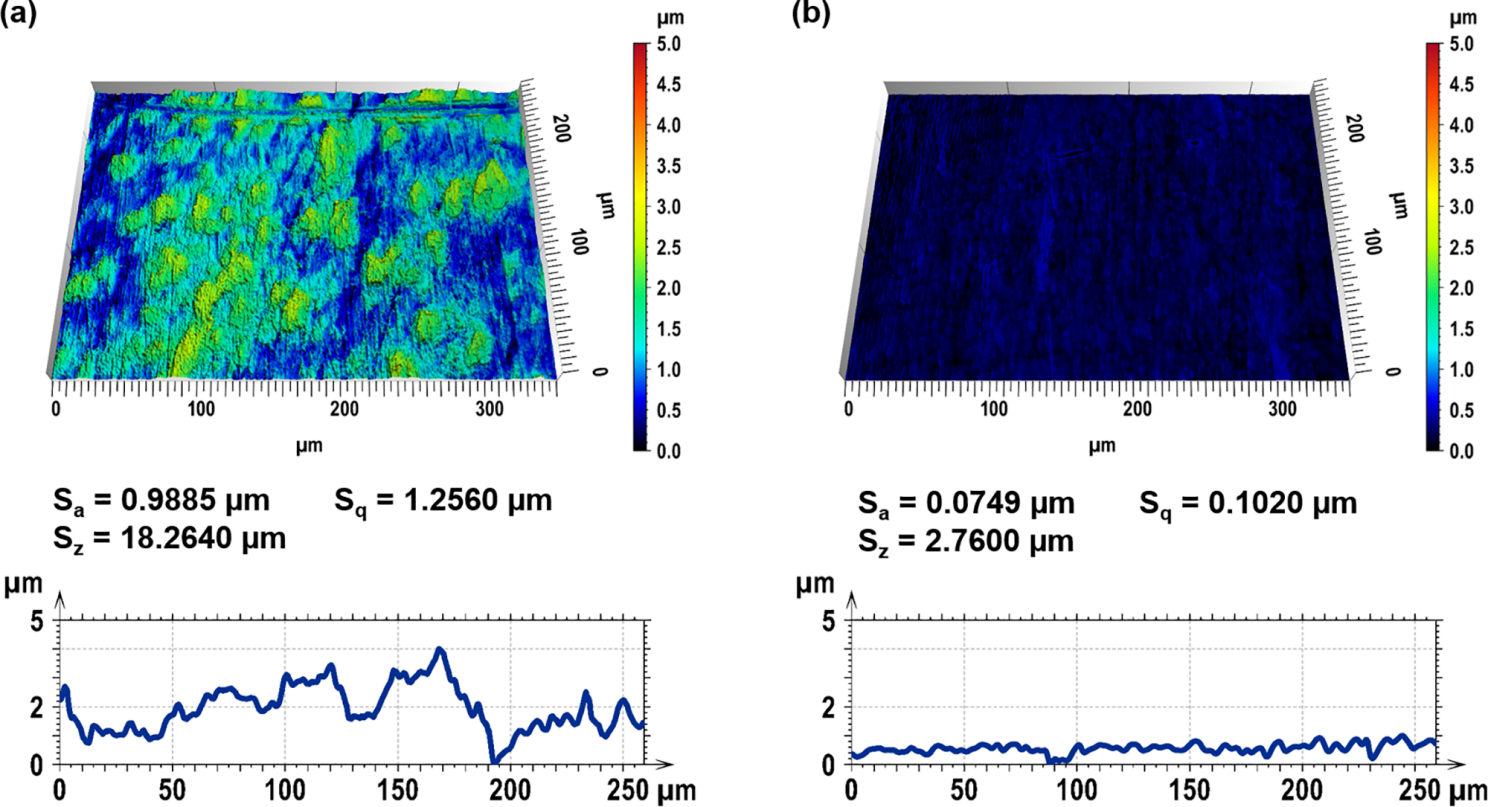
3D confocal image and 2D profile of (a) untreated Ti64 and (b) polished Ti64.

For the experiments, metal sheets out of a titanium alloy Ti64 (Titanium 6Al-4V, 90 wt. % titanium, 6 wt. % aluminum, 4 wt. % vanadium, Goodfellow) with a thickness of 1.0 mm were used as substrates. Prior to the laser texturing experiments, the specimens were laser-cut into 10 × 10 mm^2^ samples and subsequently polished with a 1200 grinding paper. Figure 1 illustrates the change in surface topography and the surface texture parameters *S*_a_ (arithmetical mean height), *S*_q_ (root-mean-square height), and *S*_*z*_ (maximum height) as a result of the polishing process.

After the DLIP functionalization, the samples were stored in centrifugal tubes (15 mL, polypropylene, LABSOLUTE), one batch with deionized (DI) water, and one batch without.

### 2.2 Direct Laser Interference Patterning

**Figure 2:**
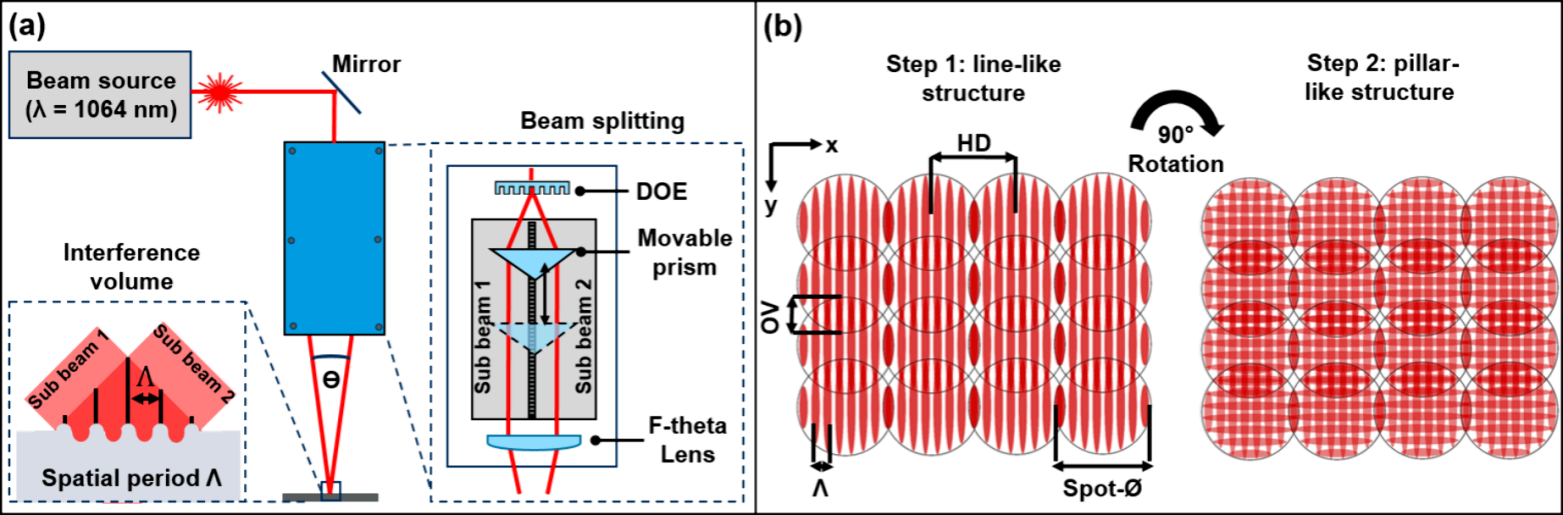
(a) Optical configuration used for laser texturing experiments and the resulting interference volume illustrating the characteristic intensity distribution for two beam interference. (b) Structuring strategy for the fabrication of pillar-like DLIP features.

The DLIP structuring experiments were performed using the optical configuration illustrated in Figure 2 a. The experimental setup consists of a solid-state laser (SN1168, EdgeWave GmbH) operating at a laser wavelength of 1064 nm with a pulse duration of 12 ps and a repetition rate of 10 kHz. For the DLIP process, the initial laser beam was split into two separated sub-beams using a diffractive optical element (DOE). The individual beams were subsequently parallelized by a prism and overlapped on the Ti64 sample under a specific incidence angle θ, generating a spot diameter of 95.0 μm. In the region of superimposition, an interference volume is formed with a line-like distribution of laser intensity. Using this optical configuration, an incidence angle θ between the overlapping beams of 10.3° is obtained. Taking into account this angle and the applied laser wavelength λ, the spatial period of the interference pattern can be calculated by using eq 1:
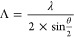
1

As a result, the overlapped sub-beams produced an interference pattern, consisting of periodically distributed lines with a spatial period of 6.0 μm. During processing, the circular laser spot was translated along the direction of the interference lines by moving the samples in two dimensions with a two-axis-positioning system (PRO155-05, Aerotech). For all laser texturing experiments, a fixed laser fluence (for each pulse) of 0.6 J cm^–2^ was used. To fabricate periodic microstructures with different surface morphologies, the pulse overlap (OV) between two individual laser spots was varied to 90 and 95% and resulted in cumulated laser fluences Φ_cum_ of 5.7 and 14.3 J cm^–2^. While changing the OV, the hatch distance (HD) was kept constant at 72 μm for the fabrication of fully textured surfaces. A pillar-like geometry was obtained by rotating a sample with a line-like pattern by 90° and irradiating again by applying the same process parameters, schematically shown in Figure 2b.

### 2.3 Surface Characterization Methods

For analysis of the surface topography of the laser-structured samples, confocal microscopy images (S-Neox, Sensofar) were recorded. The surface profiles and average structure depth were obtained using SensoMAP Advanced Analysis Software (Sensofar). In addition, scanning electron microscopy (SEM) was employed (Zeiss Supra 40VP, Carl Zeiss) for a more detailed analysis of the surface topography.

The wettability characterization was performed by static water contact angle (WCA) measurements with a contact angle measurement system (OCA 200, DataPhysics Instruments GmbH). Each measurement was executed at least three times with a droplet volume of 2 μL under ambient conditions (22 °C, 40% humidity, 1003 hPa). The standard deviation of the repetition measurements is used to describe the measurement error. Prior to the measurements, the samples that were stored in DI water were dried with a jet of pressurized air for approximately 10 s to remove residual water.

The XPS measurements were conducted using a PHI 5000 VersaProbe II system with monochromatic Al Kα as source. The measurement was performed in a 45° angle, and an area of 400 × 400 μm^2^ was analyzed. Hereby, the photons from the X-ray source get adsorbed by core electrons, causing them to be transported to the surface and to be emitted as photoelectrons with a kinetic energy equal to their former binding energy. This characteristic energy value can be used to identify particular elemental species.^30^

### 2.4 Bubble Nucleation Measurements and Image Analysis

**Figure 3:**
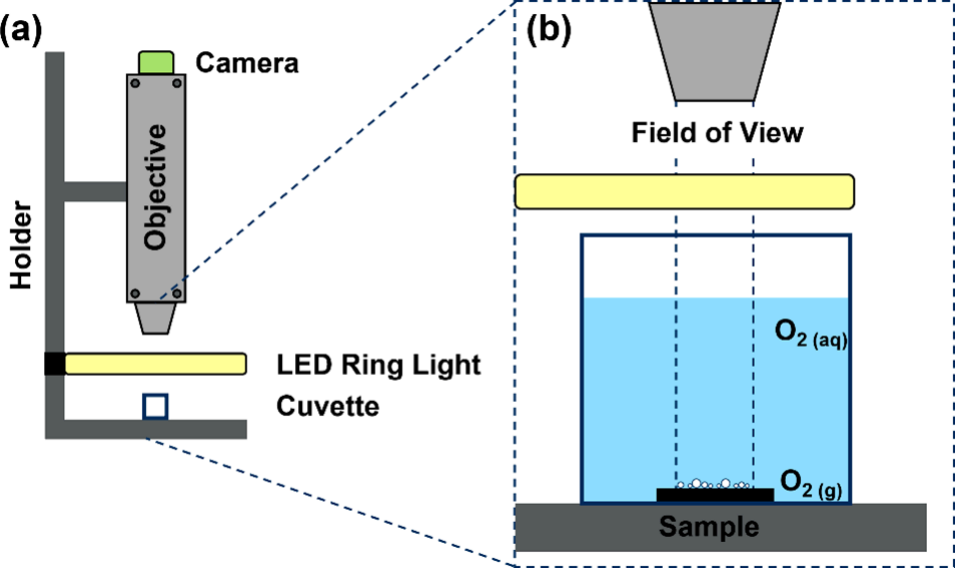
(a) Schematic side view drawing of bubble nucleation measurements setup. (b) Enlarged view of highlighted area illustrating the measurement zone and key parameters.

The exact conditions of the anodic cycle in a PEM electrolyzer stack with regard to temperature (50–80 °C), pressure (1–30 bar),^31,32^ liquid flow speed (up to multiple m/s), and gas fraction (up to 60%) are difficult to reproduce in a laboratory-scale experiment and might lead to additional side effects, e.g., from temperature gradients. Instead, a rather basic setup was employed for first model experiments with optical access, as illustrated in Figure 3a. This allowed a quick and reproducible measurement of the nucleation of the O_2_ bubbles on different surface-functionalized samples. Hereby, the sample was placed in a cuvette (704.001-OG, Hellma) and fixed to the bottom. The cuvette was placed below a three-piece objective (35-08-70-000, 35-41-10-000, 35-03-10-000, Optem) for magnification with an attached camera (GO-5100M-USB, Jai). As illumination source, a LED ring light (HPR2-150SW, CCS Inc.) connected to a digital control unit (PD3-5024-4-EI(A), CCS Inc.) was used to provide a free optical path and a partly sideways illumination for a better optical quality.

For the nucleation tests, an O_2_-oversaturated DI water mixture was used. It was freshly prepared before each measurement with an aluminum bottle (9393, BGS technic) as pressurized vessel. The solution was flushed several times with pure O_2_ (99.998% purity, Air Liquide, detailed procedure described in the SI) to purge it of any other gases and to ensure that the nucleating bubbles will mainly consist of O_2_. The final absolute pressure for the preparation of the O_2_-oversaturated DI water mixture was 1.75 bar.

Afterward, the pressurized vessel was disconnected from the O_2_ pressure tank. A tube (1 mm inner diameter) was connected to the outlet valve. The tube was fixed close to the bottom of the cuvette with the sample, and the release valve was slowly opened for a gentle inflow of the liquid. Over a period of 40 s, the vessel got filled until the water reached a filling level of 2.5 cm. Afterward, a waiting time was allowed until the liquid reached a quiescent state and the focus was adjusted. The duration between the start of the cuvette filling and the start of the recording was approximately 90 s. The bubble nucleation and growth was recorded up to 900 s with a frame rate of 0.1 fps.

The solubility of O_2_ in pure water at 25 °C and 1 bar absolute pressure is given as 1.18–1.25 mmol per liter,^33^ which equals a concentration of 37.76–40.00 mg per liter, respectively. Concentration measurements in the optical cuvette were performed with an oxygen sensor (OXYBase WR-Blue, PreSens). After the recording time of 900 s, O_2_ concentrations of ≈36 mg per liter were measured, indicating that the dissolved oxygen in the solution was still in the order of maximum solubility at atmospheric pressure. Further information on the O_2_ concentration and mass transfer can be found in the SI.

For the analysis of the grayscale images, taken with 8 bits in TIFF format, Matlab R2022b was used. Hereby, the images were analyzed regarding the circle-shaped forms of the bubbles. From the obtained data, the bubble number density, the average radius, and the visual surface coverage were determined. The visual surface coverage corresponds to the area of the image that is covered by the full diameter of the bubbles. It has to be noted that the actual contact line of the bubbles with the substrate might be hidden by the total bubble shape.

## 3 Results and Discussion

### 3.1 DLIP Ti64

**Figure 4:**
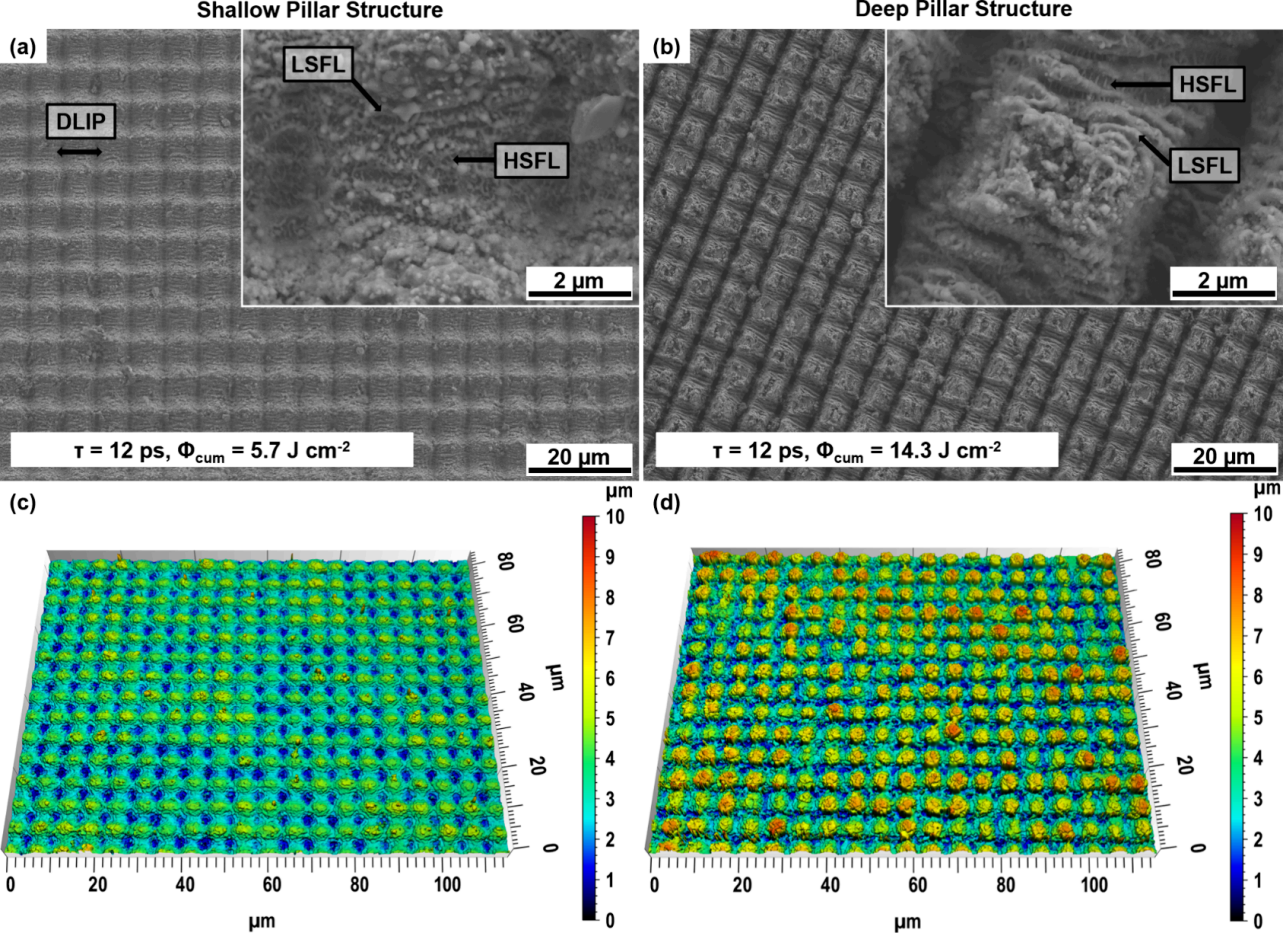
Top view—SEM images of laser-textured samples with a spatial period of 6.0 μm while applying different laser fluences of 5.7 J cm^–2^ for a shallow pillar-like texture (a), and 14.3 J cm^–2^ for a deep pillar-like texture (b) and the corresponding confocal microscopy images (c, d) of resulted Ti64 samples.

Pillar-like multiscale structures were fabricated on polished Ti64 specimens using the DLIP technique in conjunction with ultrashort laser pulses. The resulting SEM images after the laser modification of Ti64 are displayed in Figure 4a,b. In addition to the pillar-like DLIP features (Λ = 6.0 μm), smaller ripple-like surface features could be observed at the position of maximum interference. The measured periodicity of the ripples ranges from 780 to 930 nm, which corresponds to 73 to 87% of the applied laser wavelength (1064 nm). This indicates that the substructure can be regarded as laser-induced periodic surface structure (LIPSS) and further classified as low-spatial frequency LIPSS (LSFL), as reported by Bonse et al. previously.^34^ When examining the magnified SEM inset in Figure 4a,b in between the ridges of adjacent LSFL, a quasi-periodic substructure with a spatial period of approximately ∼350 nm can also be observed. This nanoripple texture can be regarded as high spatial frequency LIPSS (HSFL), as it has a periodicity smaller than half of the laser wavelength.^34^ Interestingly, these HSFL were generated on the sidewalls of the pillar DLIP features and in the areas of maximum interference. For determining the resulting structure depth of both surface morphologies, confocal microscopy was employed, shown in Figure 4c,d. The resulting structure depth was 1.7 ± 0.4 μm for the shallow pillar-like structure and 4.3 ± 1.1 μm for the deep pillar-like structure, denoted as 2 and 5 μm structure depths in the following.

### 3.2 Wettability Analysis

After functionalization, the Ti64 samples were stored in ambient air or DI water and the wettability was measured over a period of 50 days. Furthermore, the non-textured reference samples, which were stored in ambient conditions and DI water, depict WCA values of 94.40 ± 1.62 and 62.43 ± 1.32°, respectively.

**Figure 5:**
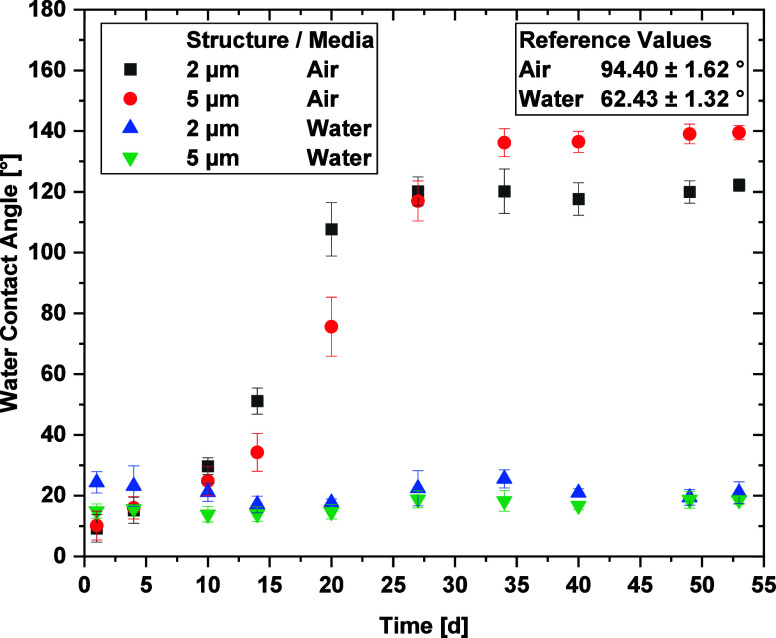
Evolution of the WCA of laser-textured Ti64 samples with different structure depths of 2 and 5 μm and different storing conditions over a duration of 50 days. The WCA of the unstructured reference samples is given as an inset.

During the DLIP structuring, the heat input causes a strong oxidation of the surface, resulting in the formation of a highly hydrophilic metal oxide layer directly after the process. Depending on the surrounding media, the samples undergo various aging processes that affect their wettability, as displayed in Figure 5. The WCA of the DI water-immersed samples remained relatively stable at 15–20°. For the samples stored in air, a continuous WCA increase to more than 120° was noticeable. The 5 μm structured samples took more time to reach their maximum of 145° compared to the 2 μm structures, while the latter only reached 125° as a stable and final value. The reason for the stronger increase in WCA can be attributed to the deeper DLIP texture combined with the formation of more pronounced LIPSS features.^26^

This aging or ripening effect of laser-structured metal surfaces, evident in their wetting behavior, is a well-known process and often reported and can mainly be attributed to the adsorption of carbon compounds from the ambient air and the increase of carbon content on the surface, based on the surface oxide layer generated before during the DLIP process.^35−38^ Nevertheless, different explanation attempts exist regarding the aging process of laser-structured metal surfaces and it can be assumed that different mechanisms take place depending on the substrate material. Kietzig et al. related the hydrophobization of their stainless steel samples to the reduction of CO_2_ to CO and zero valence carbon. Hereby, oxygen anions are transferred into vacancies of the stainless steel substrate, resulting in a Fe_3_O_4_ metal oxide layer while leaving C on the surface.^39^ On the contrary, Long et al. attributed this surface aging to the adsorption of organic matter and specifically linked the hydrophobization to the relative amount of C–C/C–H bonds on the surface.^40^ The highly polar surface and its high free energy directly after the laser structuring process cause the hydroxylation of the surface by adsorption and dissociation of water molecules from moisture in the ambient air to the unsaturated elements like Ti^4+^. These hydroxyl groups act as primary binding sites for the chemisorption of nonpolar groups.^40^ Contrary to Kietzig’s explanation, Long et al.^41^ reported a slower hydrophobization for laser-structured copper samples, which were stored in a CO_2_-rich atmosphere compared to ones that were in an organic-rich environment. Here, the amount of carbon on the surface was also identified as a critical factor.^41^

**Figure 6:**
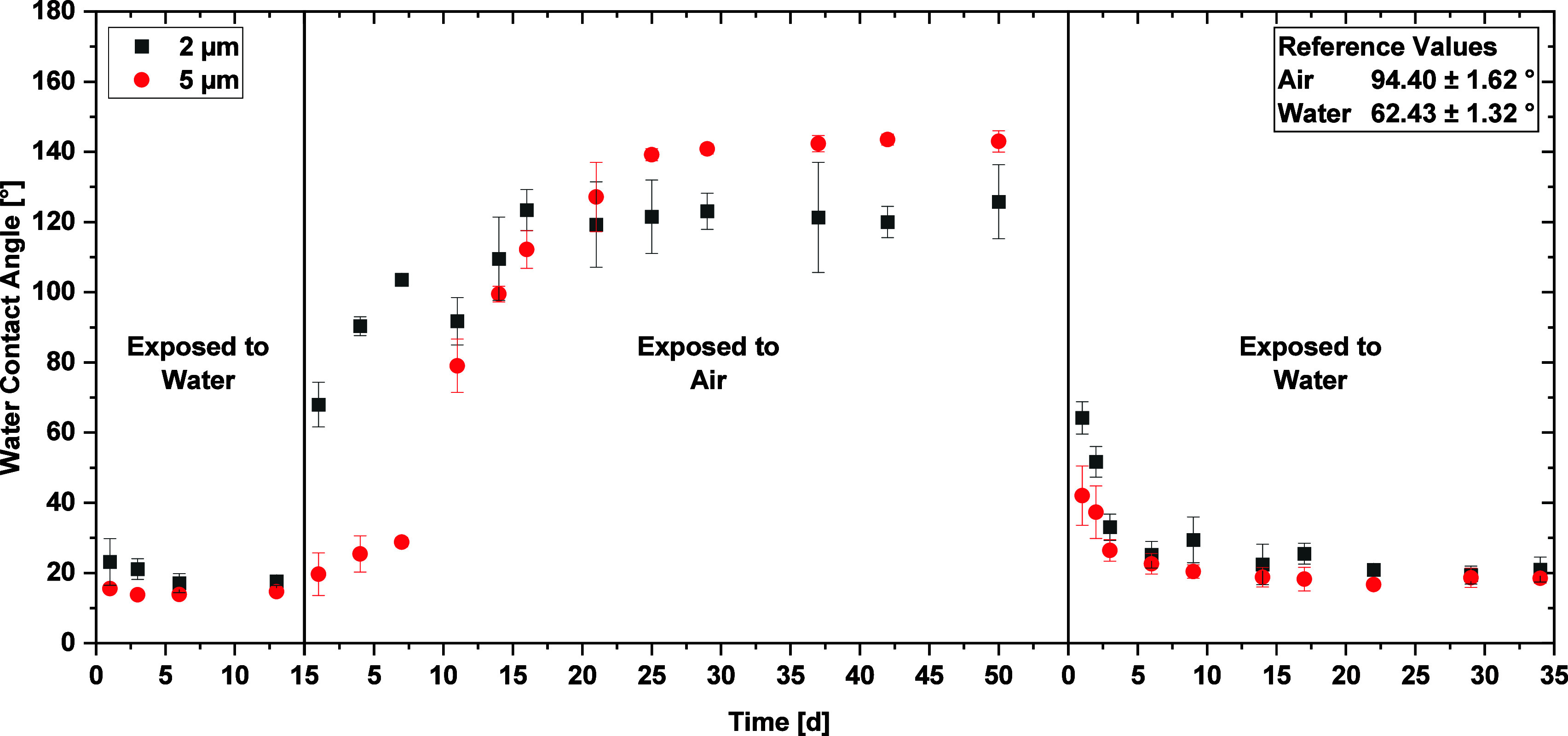
Reversibility of the ripening process with respect to the water contact angle for 2 and 5 μm-depth structured Ti64 samples. The WCA of the unstructured reference samples is given as an inset.

Furthermore, it was found that the aging of the DLIP-structured Ti64 samples is a reversible process, as shown in Figure 6. A batch of picosecond laser-structured Ti64 plates was first immersed into water, remaining at a hydrophilic wetting state with a WCA of ∼20°. Once the medium was changed to ambient air, the previously described hydrophobization process took effect and the DLIP structure with a depth of 5 μm reached a WCA of 145°. In comparison, the DLIP pattern with a structure depth of 2 μm reached their maximum WCA in a shorter time period but did not exceed 120°. These differences can be attributed to the more developed oxidation layer generated during the DLIP process for the deeper pillar-like textures, which are able to adsorb more carbon compounds, as later shown in the XPS analysis. As soon as the samples were immersed into DI water again, a drop of the WCA back to 20 °C within a few days for both structural types was observed. This indicates that the surface wettability of laser-treated substrates will always adapt to the surrounding ambient conditions.

**Figure 7:**
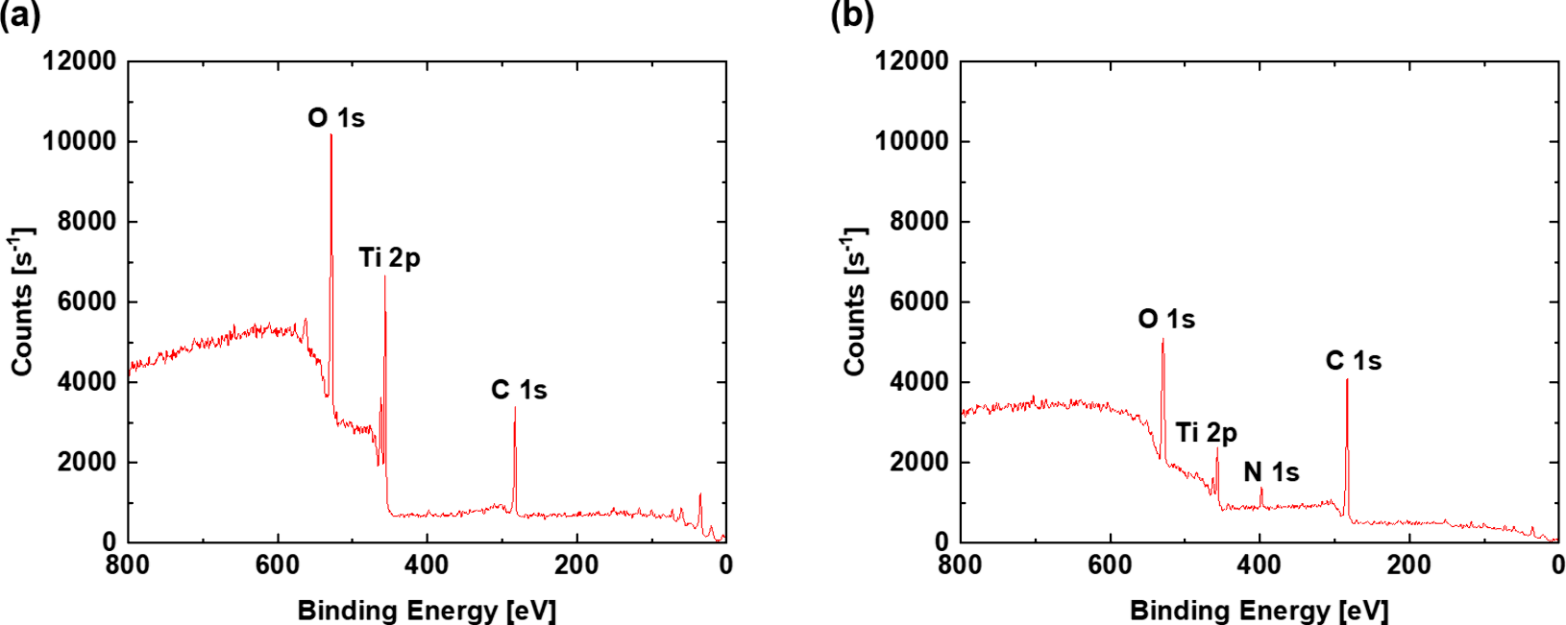
XPS survey scan with the number of electrons detected at specific binding energies for (a) reference sample and (b) 5 μm deep structured (air) sample.

A deeper insight can be gained from XPS measurements. The XPS measurements were performed after the WCA measurements shown in Figure 5 were completed. The comprehensive XPS data are contained in the SI. As listed in Table 1, the main elements found were carbon and oxygen for all samples. For the polished reference (stored on air), the percentage of carbon is the lowest in comparison to the laser-structured ones. Therefore, a high amount of oxygen and titanium is present, indicating a removal of contaminants from the surface during the polishing and later a weaker adsorption of organic compounds from the storage media in favor of an enhanced oxidation. This is also shown in the comparison of the XPS survey scans of the reference and 5 μm structured air-stored sample in Figure 7. Regarding the laser-structured samples, it can be seen that all of them feature a high carbon concentration, indicating that all are susceptible to the adsorption of compounds on the surface without dependence on the storage media. Nevertheless, the air-stored samples feature a slightly higher carbon concentration, with the 5 μm structured sample having the highest with 64.7%. On the contrary, the samples stored in water demonstrate a slightly higher oxygen amount. All of the laser-structured samples also contain a certain amount of nitrogen, which most likely originates from the adsorbed organic compounds. All surfaces also show negligible amounts of F, Na, Si, S, Cl, and K.

**Table 1:** Surface Composition (at. %) Determined by Fitting XPS Data

element	C (at. %)	O (at. %)	Ti (at. %)	Al (at. %)	V (at. %)	N (at. %)
reference	39.6	42.3	13.3	3.6	0.4	0.7
2 μm (air)	60.8	28.0	2.7	1.2	0.0	7.3
5 μm (air)	64.7	24.8	3.9	2.1	0.1	4.4
2 μm (water)	58.6	28.8	2.9	1.2	0.1	8.3
5 μm (water)	48.9	37.1	5.9	2.4	0.1	5.6

While all the laser-structured samples show clear differences regarding the surface composition to the reference sample, the different at. % of the elements between those samples does not directly explain their different wetting behavior.

**Figure 8:**
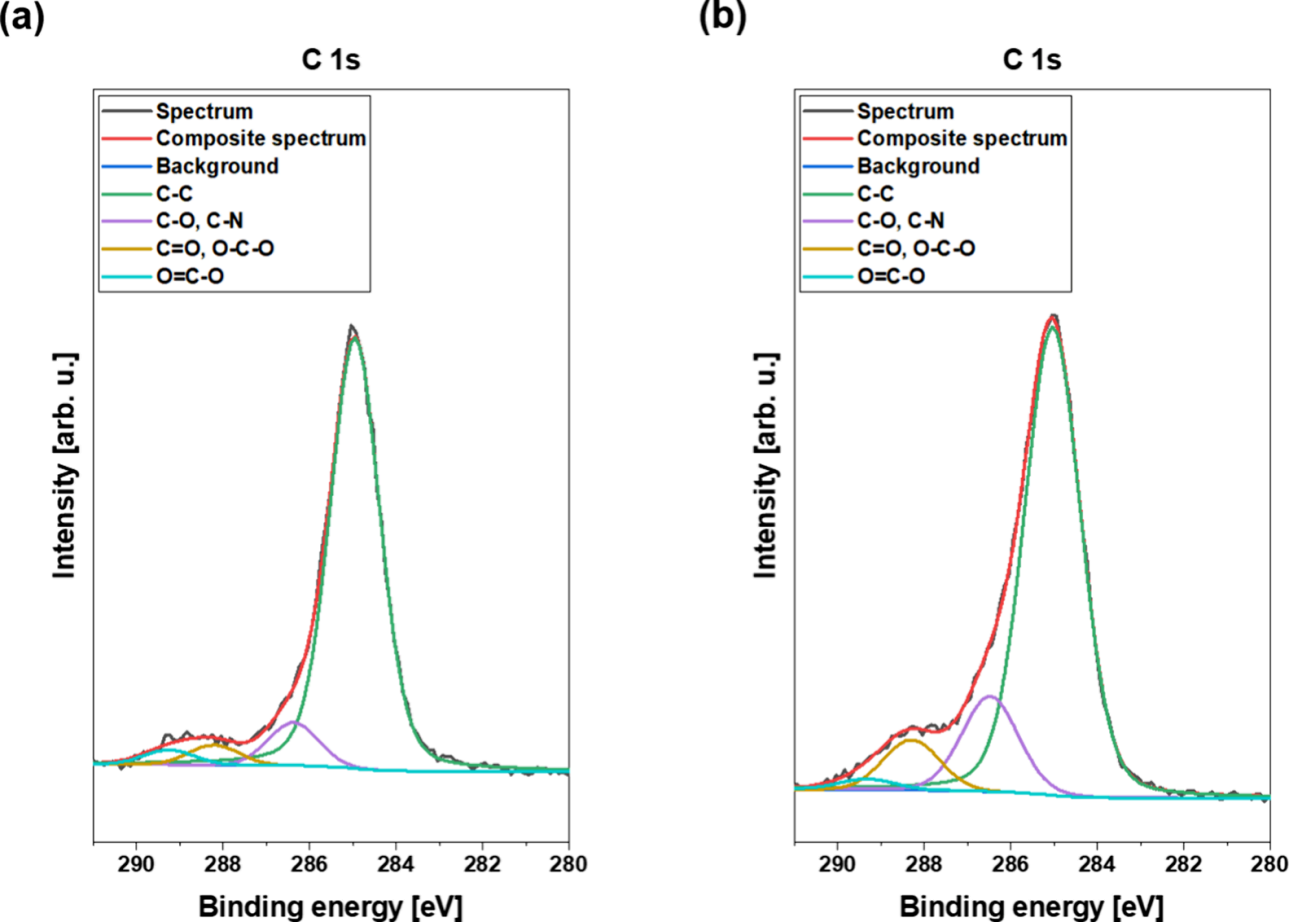
C 1s high-resolution XPS spectra for the (a) reference sample and (b) 5 μm (air) sample.

The detailed C 1s XPS spectra for the reference and the 5 μm air-stored sample are shown in Figure 8a,b. In general, the red curve shows the composite spectrum, which corresponds to the electron configuration of the respective atom and can be related to the amount of the corresponding element within the sampling volume. This composite spectrum in turn consists of several subspectra, which allow to evaluate the prevailing chemical bonds and groups. The reference features only small peaks at 286.5 and 288.3 eV for carbon–oxygen (C–O) bonds, carbon–nitrogen (C–N) bonds, carbonyl (C=O) bonds, and O–C–O bonds, as seen in Figure 8a. On the contrary, all the laser-structured samples, as shown in Figure 8b, have much more pronounced signals for these bonds, especially regarding the C–N one, confirming a strong adsorption of organic compounds and giving an explanation for the high amount of nitrogen detected. However, from all the listed carbon bonds in Table 2, only the C–C and the C–N bonds can be characterized as nonpolar and therefore contribute to a decreased wettability and an increased WCA. All samples show a similar peak around 285.0 eV for the carbon–carbon (C–C) bond with a percentage of roughly 30%, as listed in Table 2. An exception hereby is the 5 μm air-stored sample with 49.2%, which therefore explains why the sample nearly reached superhydrophobic conditions with a WCA of 145°.

**Table 2:** Detailed Surface Composition (at. %) for C 1s and O 1s

	C 1s				O 1s		
energy [eV]	285.0	286.5	288.3	289.3	530.2	531.8	532.7
							
groups	C–C	C–O	C=O	O–C=O	O–Ti	O=C	O–C
		C–N	O–C–O		O–Al	O–Si	–OH
							
reference	34.1	3.0	1.5	1.0	29.2	6.7	6.4
2 μm (air)	34.3	17.2	7.9	1.4	8.0	16.9	3.1
5 μm (air)	49.2	9.5	4.8	1.1	10.5	7.7	6.6
2 μm (water)	33.0	16.2	8.6	0.8	8.8	12.0	7.9
5 μm (water)	24.6	15.9	7.0	1.5	16.4	9.0	11.7
							
chemical compound	contaminations				oxides, hydroxides, contaminations		

Furthermore, at 530.2 eV, the reference sample features a strong peak, indicating a higher amount of metal oxides in the form of O–Ti and O–Al due to less adsorption and a stronger exposition to oxidation compared to the laser-structured samples.

Based on the XPS analysis, it can be seen that the DI water-stored samples feature a slightly higher at. % for oxygen, with 28.0% for the 2 μm structured sample when stored on air and 28.8% when stored in water, and 24.8% for the 5 μm structured sample when stored on air and 37.1% when stored in water. Also, the C–C peak at 285.0 eV is lower for the water-stored samples while the O 1s region shows higher peaks at 532.7 eV, indicating a higher amount of hydroxyl groups, which presumably are the reason for the increased wettability. Based on these results, it can be assumed that an adsorption of organic compounds takes place for all DLIP-functionalized samples. The major difference of the water-storage consists of the additional introduction of hydroxyl groups, causing a decrease in WCA and an increase in wettability. In summary, the XPS results explain the hydrophobization effect on air and the hydrophilization effect for water-stored samples. Defined surface roughness further amplifies the hydrophobization and hydrophilization effect,^42,43^ leading to the observed strong differences in WCA for the DLIP-functionalized samples. From the results listed above, it can be concluded that the DLIP approach changes wettability through a modification of surface chemistry and topography.^44^

### 3.3 Oxygen Bubble Nucleation

**Figure 9:**
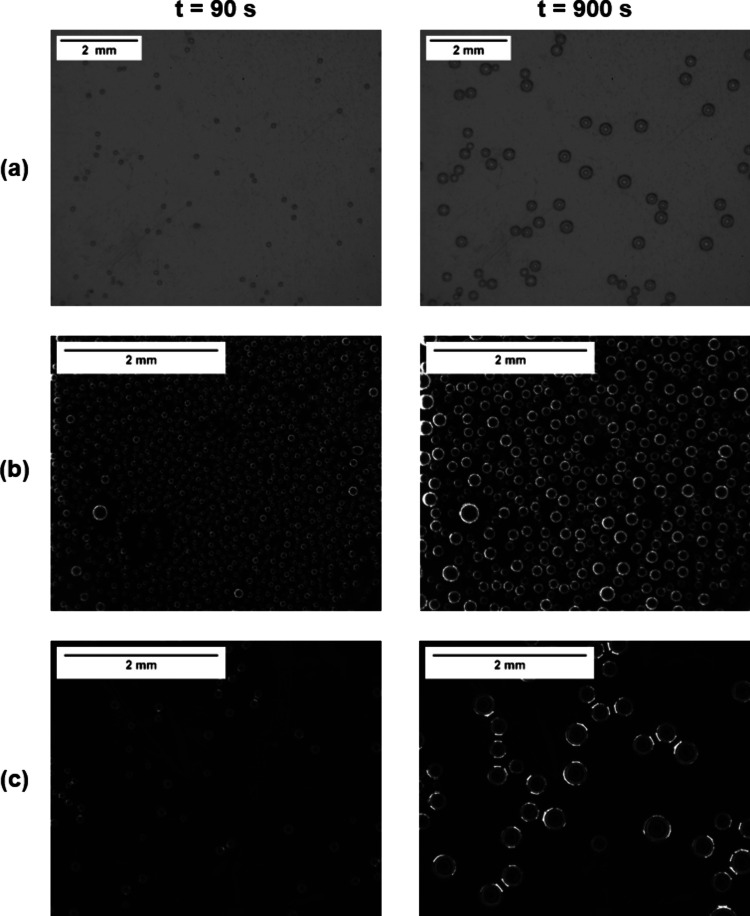
Example images of O_2_ bubbles nucleated on the different substrates for *t* = 90 and *t* = 900 s: (a) reference; (b) 5 μm air-stored; (c) 5 μm DI water-stored.

The analysis of the O_2_ bubble nucleation was done for 5 μm DLIP-structured samples, since those feature the highest and lowest wettability, respectively, as shown previously in Figure 5. Also, the Ti64 reference (air-stored) was analyzed for comparison. For each sample type, three repeated bubble nucleation tests were used for the analysis. Example images of the three different sample types can be seen in Figure 9, showing the nucleation of the O_2_ bubbles at 90 s and at 900 s after immersion into the liquid.

**Figure 10:**
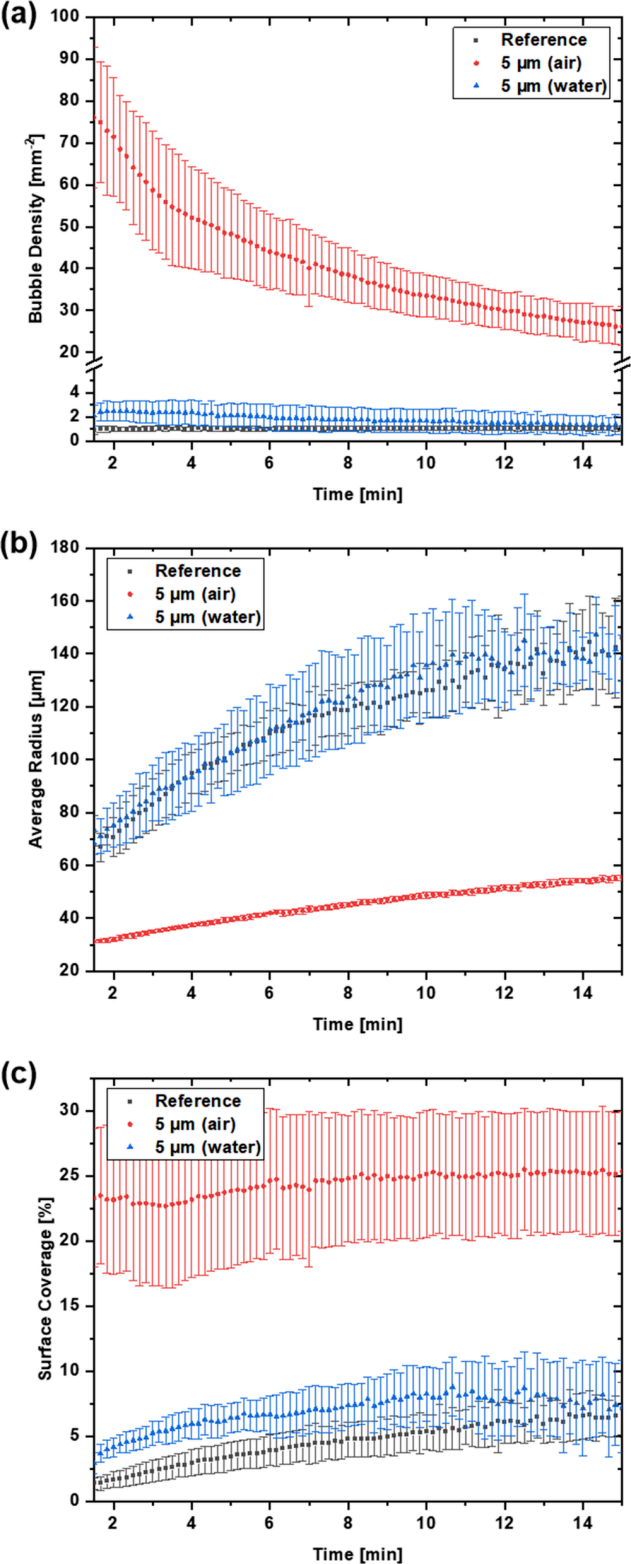
(a) Bubble density, (b) average bubble radius and (c) surface coverage against the measurement time for the Ti64 reference and the 5 μm ps laser-structured samples, which were stored in DI water or on air beforehand.

In principle, increased nucleation can be expected for hydrophobic surfaces due to the higher void probability and the preferred interactions of the solid with the hydrophobic gas molecules. Despite having the most hydrophilic character, the 5 μm laser-structured sample features a slightly higher bubble density per mm^2^ compared to the reference, as displayed in Figure 10a. These observations can be attributed to the surface enlargement by the DLIP microstructures, which introduce more cavities and nucleation sites compared to the reference sample. A comparable trend can also be seen from the curves for bubble growth and surface coverage (seen in Figure 10b,c), indicating similar bubble dynamics for the reference and the 5 μm water-stored Ti64, despite the enhanced wettability for the latter one. For both samples, the bubble growth is mainly based on diffusive O_2_ mass transfer from the oversaturated bulk solution. Especially for the reference, the distance between the bubbles is too large for noticeable coalescence. In the later stages of the experiment, the bubbles for the 5 μm water-stored Ti64 reach a size where the buoyancy force overcomes the relatively weak interaction forces with the surface, initiating bubble detachment. This leads to a decrease in bubble density and therefore also in surface coverage, which causes both values to approach the reference again. To validate if the decrease in bubble density is based on detachment or coalescence, difference images were created by subtraction of the previous image from the current one. These difference images yield distinct fingerprints for the specific events, i.e., simple bubble detachment (appearing as single negative bubble), detachment due to coalescence (two or more neighboring negative bubbles), and coalescence without detachment (two negative bubble parts next to a positive bubble). Hence, these images allowed to spot and to distinguish between the different events. The ratio between coalescence and detachment events gives some insight into the interaction strength between the bubbles and the surface based on its character. For the reference sample, no detachment events were noticeable and the slight decrease in bubble density is only based on very few coalescence events. For the 5 μm water-stored Ti64 samples, multiple bubbles were lost due to detachment. However, this detachment usually originated from the previous coalescence of two bubbles. Only around 25% of the merged bubbles remained on the surface after coalescence.

Significantly different dynamics are visible for 5 μm air-stored Ti64. Compared to the other samples, it features a roughly 20 times higher bubble density at the beginning of the recording, which notably decreases with time, as seen in Figure 10. Due to the higher bubble density, the average radius only slightly increases with ongoing mass transfer and the surface coverage remains relatively constant at approximately 25% for the whole recording period, which is still about 350% higher compared to the reference and the water-stored sample, with both reaching a maximum value of slightly above 7% surface coverage. This means that the majority of nucleation happens within the first 90 s for this hydrophobic surface, i.e., the major part of the dissolved O_2_ in the water has already been transferred to the gas phase in the initial stage. Consequently, the bubble growth is only based to a small degree on diffusion while coalescence has a much stronger impact. Due to the high density of bubbles, their distance to each other is significantly smaller, and hence, the coalescence probability is higher, causing the number of bubbles to decrease while the average radius increases. Only about 33% of the merged bubbles detached from the samples, while the rest remained on the surface. The detachment of single bubbles was observed only rarely. The difference in bubble detachment between the 5 μm water- and air-stored samples is due to the generally smaller bubble size and the lower buoyancy forces for the latter but also to the stronger interactions with the hydrophobic surface.

## 4 Conclusions

In summary, the change of the surface wettability after DLIP, its dependence on the chemical surface composition, and its impact on the O_2_ bubble nucleation were investigated. The following statements can be derived from this work:

- After DLIP structuring, all Ti64 samples undergo a ripening process, no matter if stored on air or in water. In both media, organic compounds from the surrounding environment are being adsorbed on the substrate surface, resulting in a strong increase of carbon by roughly 10–20 at. %.
- The hydrophobization process for deeper structures needs more time to reach its final value, and the resulting maximum WCA is also higher, probably due to the larger surface area, which is available for the adsorption of carbon compounds.
- The ripening process and the surface wettability changes are reversible and adapt to the storage media, i.e., water-stored samples become hydrophilic and air-stored samples hydrophobic. Hereby, the changes are not predominantly based on adsorption and desorption processes of organic compounds but rather by the amount of existing polar groups on the surface like hydroxyl groups.
- Highly hydrophobic surfaces strongly enhance the O_2_ bubble nucleation. Based on the higher affinity between surface and dissolved gas, a significantly quicker nucleation process takes place with an increase in the number of nucleation spots by more than 20 times. Since at the same time the bubble radius is significantly smaller, the overall surface coverage increased by approximately 350%.
- Detachment of bubbles is favored on hydrophilic substrates and usually takes place directly after the coalescence of two bubbles, with around 75% of the merged bubbles detaching afterward. Due to the stronger interaction forces, detachment is less likely even after coalescence on hydrophobic samples, with a detachment rate of approximately 33%.

It was possible to validate that the DLIP technique is a useful method to change the surface character of Ti64 and to strongly affect the O_2_ bubble nucleation. The DLIP-generated structures hereby amplify the substrate wettability changes based on the surrounding media and can be used to tune hydrophilic and hydrophobic properties. Therefore, in a technological application, deep hydrophobic structures could be used for a temporal enhancement of O_2_ nucleation. This would enable a more effective separation process of the gas from the anodic PEM cycle, and thus a more efficient startup phase before the laser-treated surface adapts to the aqueous environment and turns hydrophilic. On the contrary, also the hindrance of the nucleation by hydrophilic structures can be used purposefully, for example, in the heat exchanger, where gas surface coverage is disadvantageous. Furthermore, these surfaces would be able to retain their hydrophilic character in an aqueous environment. The associated enhanced bubble detachment from the hydrophilic metal surface is also advantageous for other parts of the electrolyzer. For example, it was demonstrated that a quicker bubble detachment based on the hydrophilicity of laser-generated structures increases the performance of the electrocatalyst surface.^45,46^

Concluding, this research shows that the DLIP technique can be used for the treatment of metal substrates to adapt their surface wettability and therefore change the interaction of dissolved gases with the surfaces in different parts of industrial facilities. Aside from the possible PEM related application, our results also provide important insights for the application of laser-based surface functionalization in other multiphase processes such as enhanced heat transfer and boiling performance^47^ as well as anti-icing effects.^26,48^

## Supporting Information Available

Comments on the O_2_-oversaturation procedure, image editing and analysis process, deconvolution and fitting of XPS spectra, supporting figures of fitted XPS spectra and image processing steps (PDF)
Supporting videos of O_2_ bubble nucleation (ZIP)
