# Supplementary material for: Functionalization of Ti64 via Direct Laser Interference Patterning and Its Influence on Wettability and Oxygen Bubble Nucleation
Source: Langmuir. 2024 Jan 31;40(6):2918–29. doi: 10.1021/acs.langmuir.3c02863 (PMC10867896; doi:10.1021/acs.langmuir.3c02863)
Supplement: Supplementary file 1 — la3c02863_si_001.pdf [file la3c02863_si_001.pdf]

Supporting Information

Functionalization of Ti64 via Direct Laser Interference Patterning and Its Influence on Wettability and Oxygen Bubble Nucleation

Julian Heinrich[1,2], Fabian Ränke[3], Karin Schwarzenberger[1,2], Xuegeng Yang[1,2], Robert Baumann[3], Mateusz Marzec[4], Andrés Fabián Lasagni[3,5], Kerstin Eckert*[1,2]*

1 Institute of Fluid Dynamics, Helmholtz-Zentrum Dresden-Rossendorf, Bautzner Landstr. 400, Dresden 01328, Germany

2 Institute of Process Engineering and Environmental Technology, Technische Universität Dresden, Helmholtzstr. 14, 01069 Dresden, Germany

3 Institute of Manufacturing Science and Engineering, Technische Universität Dresden, George-Baehr-Str. 3c, 01069 Dresden, Germany

4 Academic Centre for Materials and Nanotechnology, AGH University of Krakow, Av. Mickiewicza 30, 30-059 Krakow, Poland

5 Fraunhofer Institute for Material and Beam Technology IWS, Winterbergstraße 28, 01277 Dresden, Germany

Corresponding Authors

*j.heinrich@hzdr.de, kerstin.eckert@tu-dresden.de

Appendix A: Supporting Comments

A.1 Comment on the O_2-Oversaturation

The oversaturation was achieved by the following steps:

- Fill the pressure vessel with 350 ml DI water
- Connect it to the O_2 pressure tank
- Apply an O_2 overpressure of 3 bar to the vessel
- Shake the vessel by hand for 3 s and afterwards open the release valve for 1 s (repeat 5 times in total); afterwards let the vessel rest for 1 min
    - repeat the whole procedure 3 times in total
- Over pressure the vessel at 1.75 bar
- Shake the vessel by hand for 3 s and afterwards open the release valve for 1 s (repeat 5 times in total); let the vessel rest for 1 min
    - repeat the whole procedure 2 times in total

A.2 Comment on the O_2-Concentration and O_2-Diffusion

With the used oversaturation pressure of 1.75 bar a theoretical concentration of 66.08 – 70.00 mg per liter is expected. For further validation, the oxygen concentration was also measured in an instrumented oversaturation vessel with a value of 76 ± 10 mg per liter using three independent sensors (Memosens COS51E-1009/0, Endress+Hauser). This deviation is caused by a calibration uncertainty of the sensors, since the calibration is done by atmospheric pressure yet the measurement itself is performed at overpressure. After the recording time of 900 s, O_2 concentrations of ≈ 36 mg per liter were measured, indicating that the dissolved oxygen in the solution was still in the order of maximum solubility at atmospheric pressure.

During the recording of the bubble growth, diffusive mass transfer between the supersaturated solution and the ambient air will take place which however is assumed to be negligible, i.e. the main mass transfer processes are due to the bubble nucleation at the functionalized surfaces. This is supported by estimating the diffusion flux J of oxygen across the air-water interface via Fick's first law $J = -D \cdot \frac{\Delta c}{\Delta \mathrm{x}}$, where D is the diffusion coefficient of O_2 in water[1] and $\frac{\Delta c}{\Delta \mathrm{x}}$ an approximated concentration gradient. Hereby, the concentration difference is based on the theoretical maximum oxygen concentration of 1.25 mol per m^3 at 1 bar O_2 pressure (which can be assumed due to the previous oversaturation at 1.75 bar) and the value of 0.256 mol per m^3 for normal atmospheric conditions (equilibrium with air).[2] The layer height Δx of the aqueous solution above the functionalized surface corresponds to the filling level of 0.025 m.

The estimated loss of oxygen molecules based on diffusion across the water surface A of 0.0009 m^2 (cuvette cross section) during the recording time t of 900 s is $\Delta n = 6.7632 \cdot 10^{-8} mol$. This yields an average concentration difference of Δc = 0.003006 mol per m^3 (0.096185 mg per liter) over the whole liquid volume, which is negligible with respect to the initial concentration of 1.25 mol per m^3 (40.00 mg per liter).

A.3 Comment on the Image Editing and Analysis Process

All the different sample types generated different optical recordings regarding image quality and bubble size. Therefore, the analysis process was adapted for each sample type. For the analysis of the grey-scale images, taken with 8 bits in TIFF format, Matlab R2022b was used.

	Reference	5 µm (air)	5 µm (water)
Preprocessing	▪ Conversion into grayscale<br>▪ Image contrast adjustment	▪ Conversion into grayscale<br>▪ Image contrast adjustment<br>▪ Apply threshold<br>▪ Close circles<br>▪ Fill circles	▪ Background subtraction<br>▪ Conversion into grayscale<br>▪ Image contrast adjustment<br>▪ Apply threshold<br>▪ Close circles<br>▪ Fill circles
Circle Detection	"imfindcircles"<br>▪ ObjektPolarity = dark<br>▪ Sensitiviy = 0.900	"imfindcircles"<br>▪ ObjektPolarity = bright<br>▪ Sensitiviy = 0.875	"imfindcircles"<br>▪ ObjektPolarity = bright<br>▪ Sensitiviy = 0.925

A.4 Comment on Deconvolution and Fitting of C, O and Ti XPS Spectra

The C 1s spectra can be fitted with four components: the first line at 285.0 eV indicates the presence of aliphatic carbons, the second line C-O and/or C-N groups with the line present at 286.5 eV, the third line at 288.3 eV points out the existence of C=O and/or O-C-O type bonds and the last line at 289.3 eV originates from O-C=O type bonds.[3]

The O 1s spectra for all samples were fitted with three components: the first line centered at 530.2 eV indicates mainly metal oxides like O-Ti and O-Al, the second line at 531.8 eV results from either metal hydroxides and/or some part of O=C organic bonds and O-Si bonds and the third line at 532.7 eV comes from organic O-C and/or –OH bonds.[4]

The Ti 2p spectrum was fitted with a doublet structure ($p_{3/2}$ – $p_{1/2}$ doublet separation equals 5.7 eV) with the main $2p_{3/2}$ line centered at 458.7 eV, indicating the Ti^{4+} oxidation state.[4–6]

Appendix B: Supporting Figures

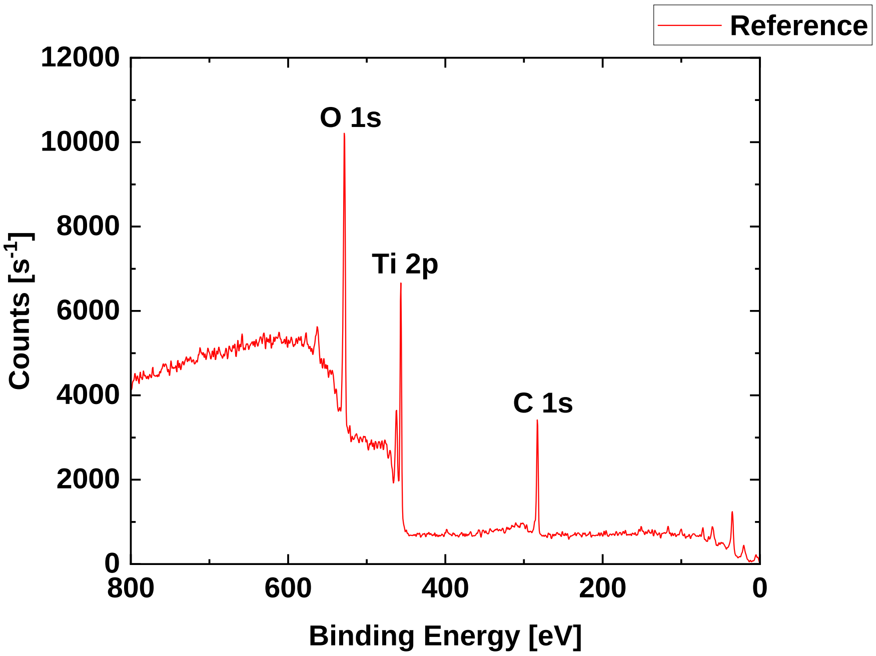

Figure 1. XPS survey scan reference sample.

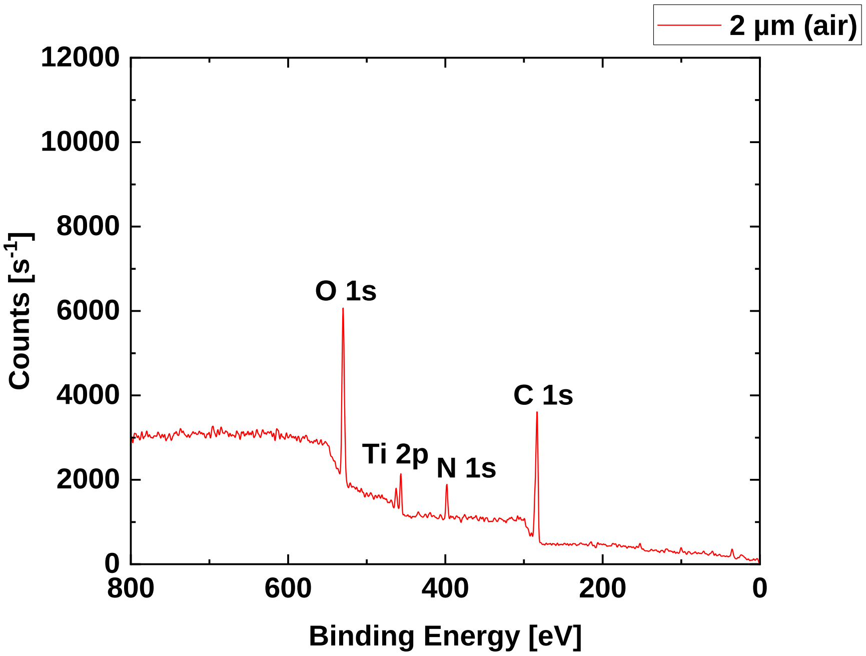

Figure 2. XPS survey scan 2 µm (air) sample.

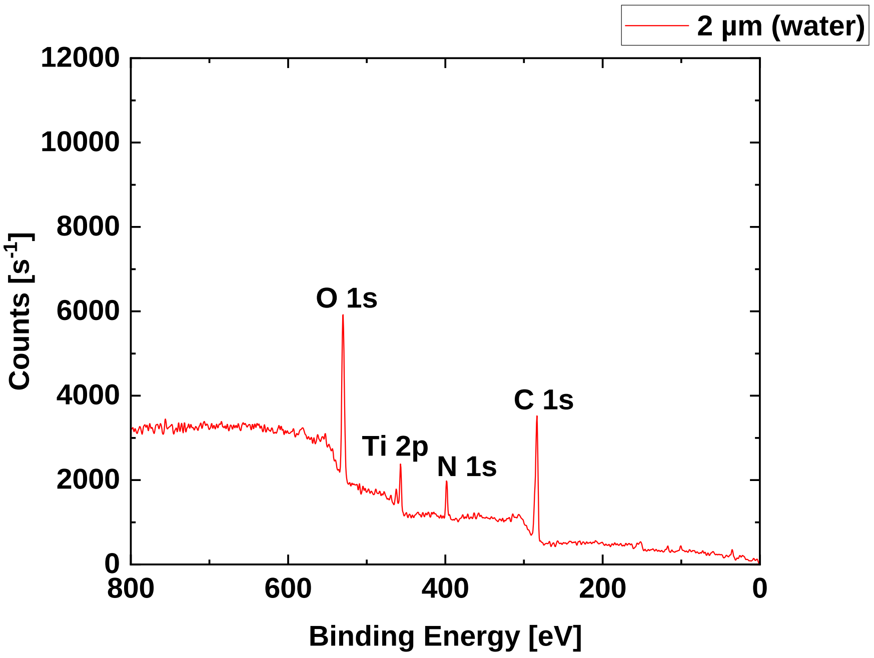

Figure 3. XPS survey scan 2 µm (water) sample.

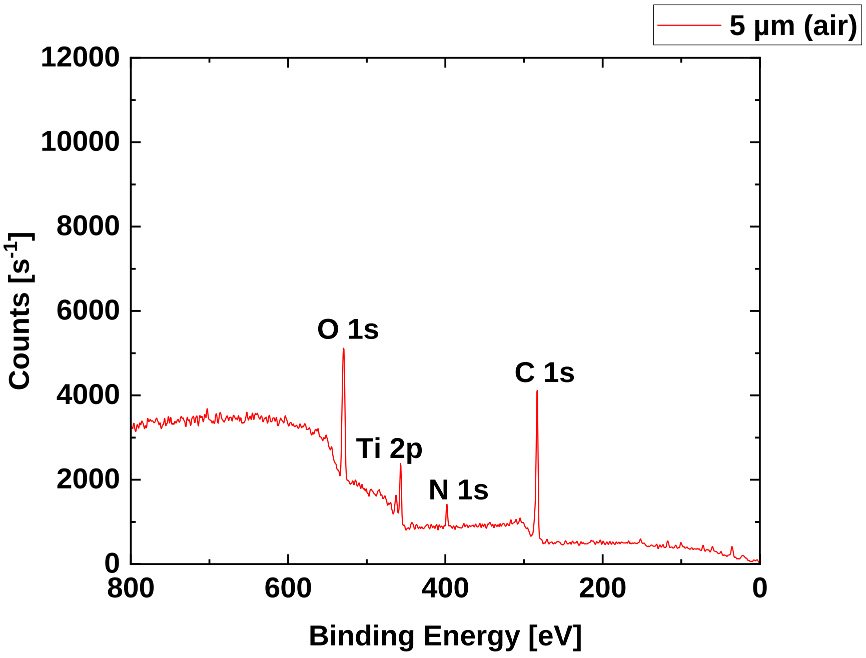

Figure 4. XPS survey scan 5 µm (air) sample.

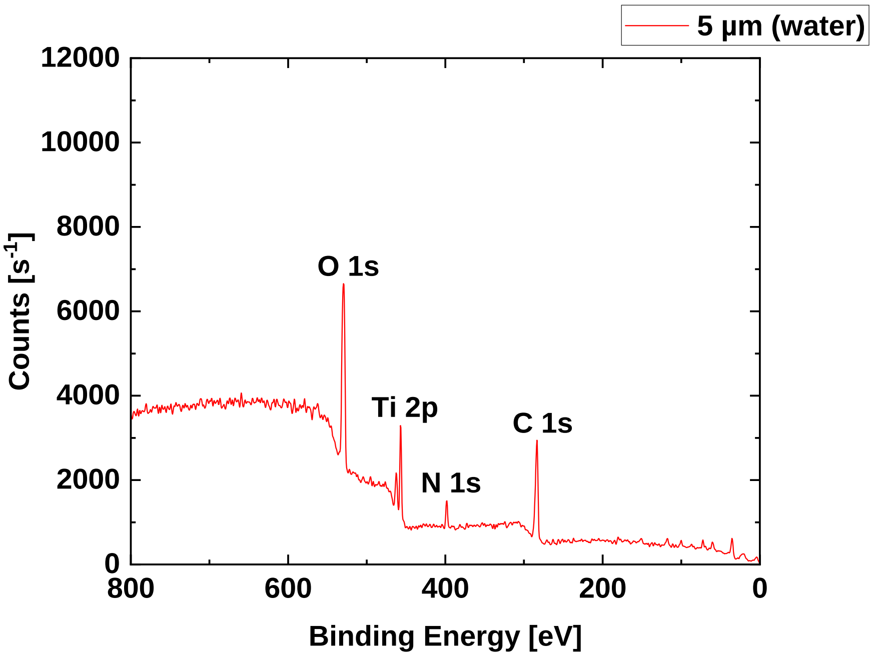

Figure 5. XPS survey scan 5 µm (water) sample.

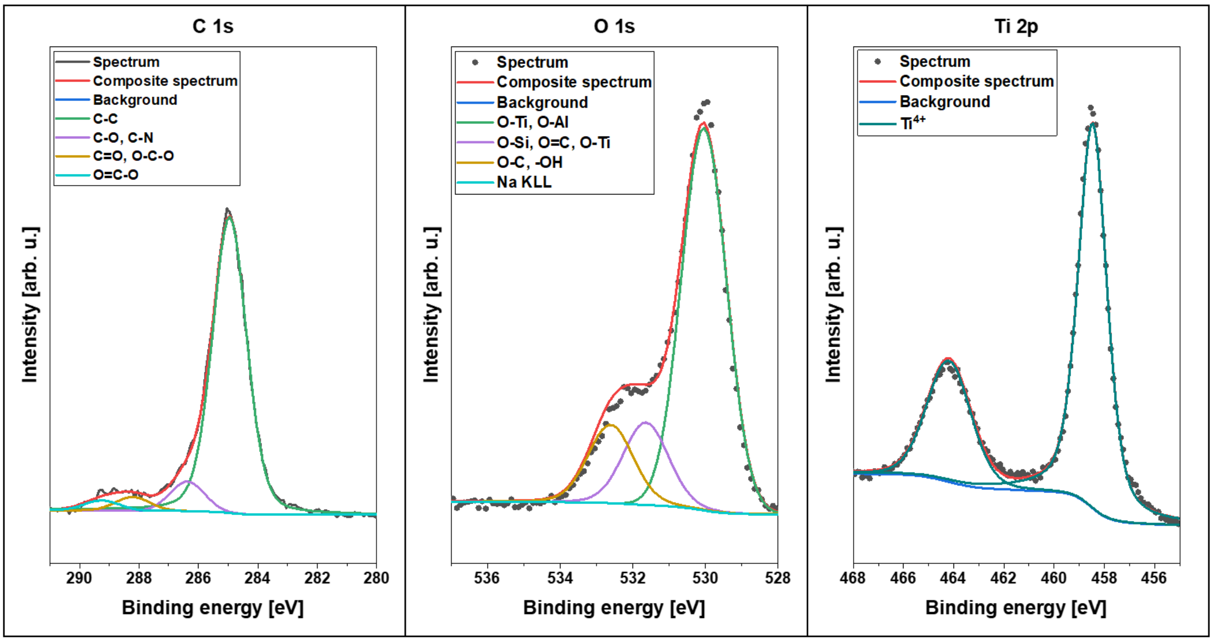

Figure 6. High resolution C 1s, O 1s and Ti 2p XPS spectra reference sample.

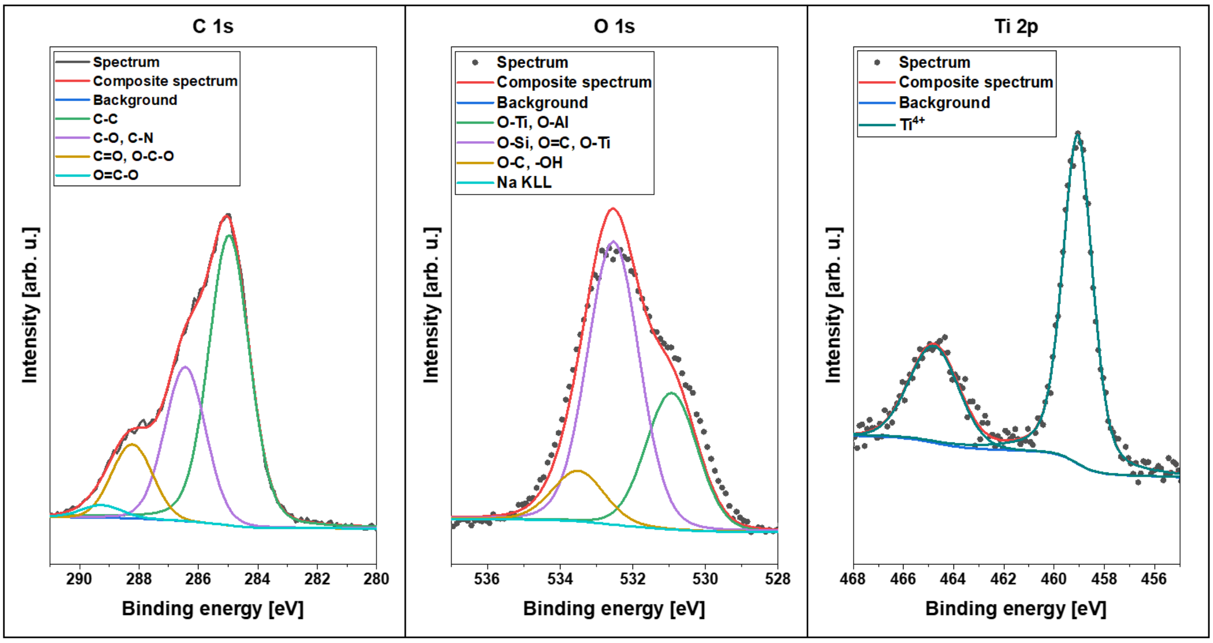

Figure 7. High resolution C 1s, O 1s and Ti 2p XPS spectra 2 µm (air).

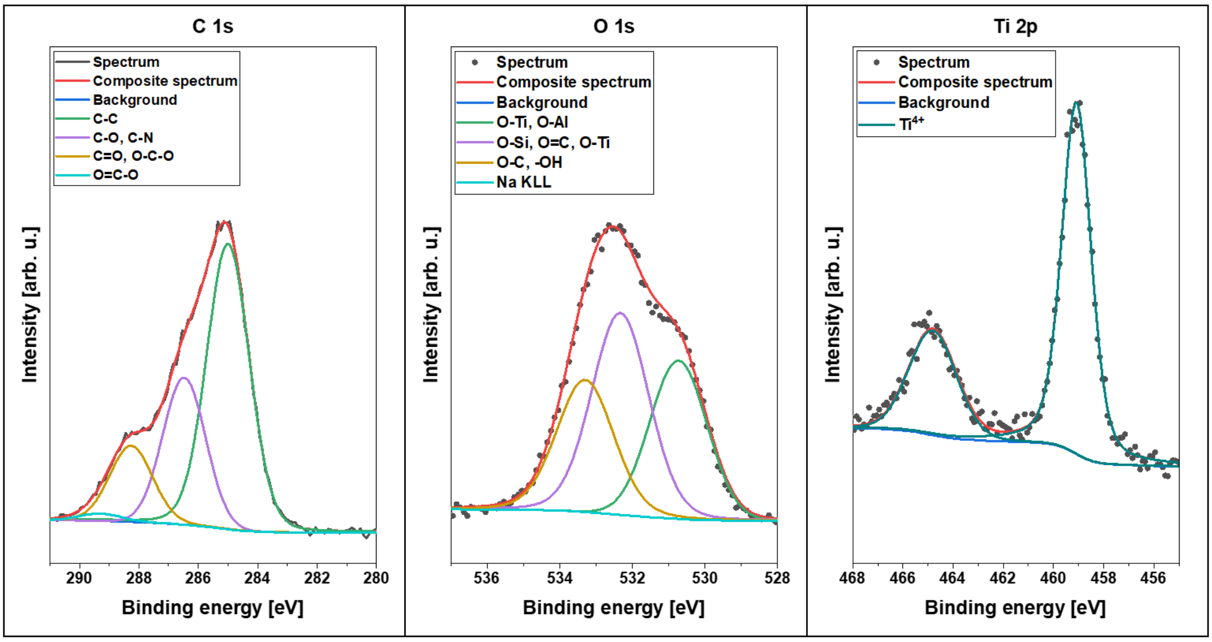

Figure 8. High resolution C 1s, O 1s and Ti 2p XPS spectra 2 µm (water).

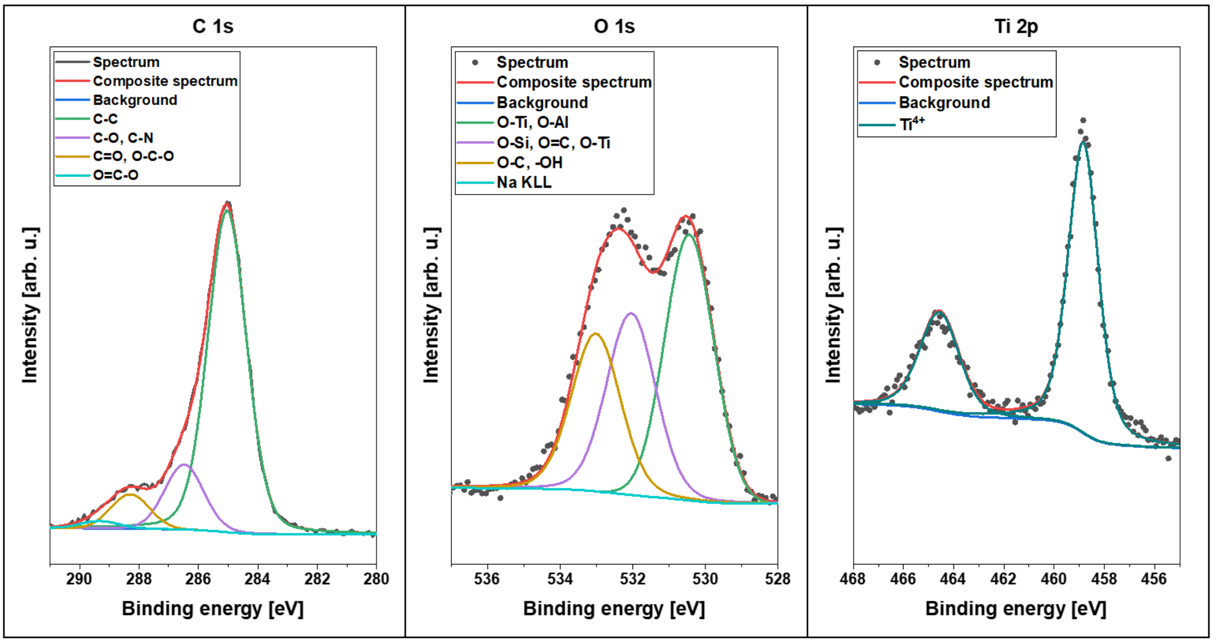

Figure 9. High resolution C 1s, O 1s and Ti 2p XPS spectra 5 μm (air).

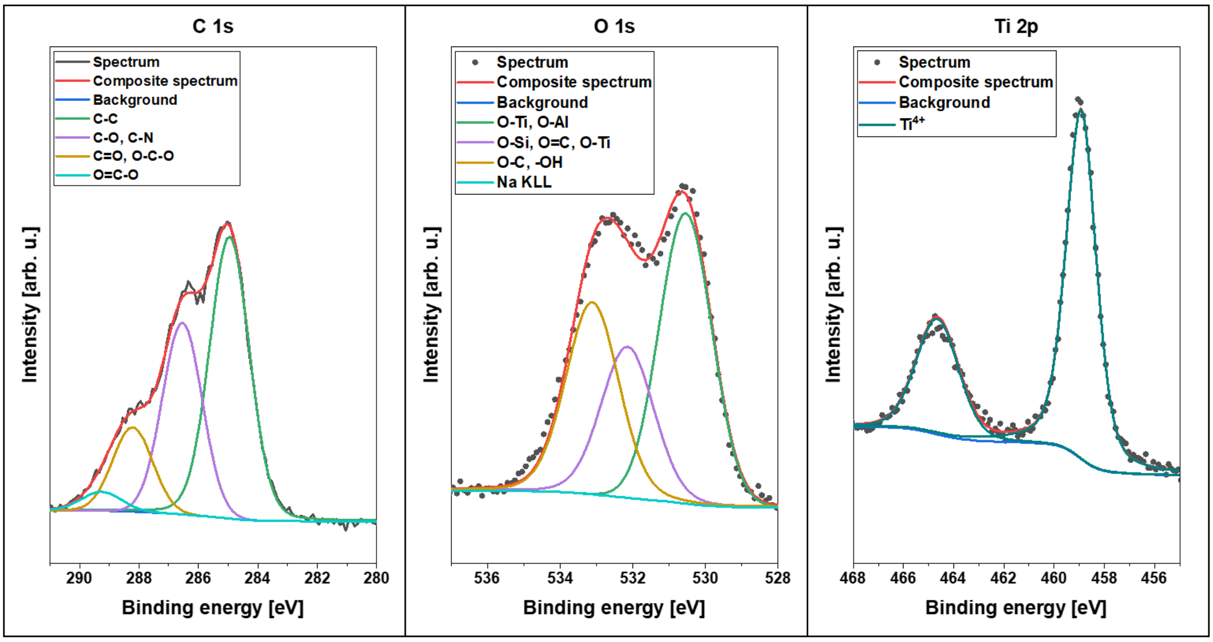

Figure 10. High resolution C 1s, O 1s and Ti 2p XPS spectra 5 μm (water).

The Ti 2p region (Fig. 6 to 10) shows a peak doublet for all samples at 458.7 eV and 465.0 eV corresponding to the Ti $2p_{3/2}$ and Ti $2p_{1/2}$ indicating an oxidation of the metal. This could again be connected to the O 1s peaks at 530.2 eV. These peaks are in general higher for the reference sample, validating again that less compounds were adsorbed on the surface and the titanium itself was more exposed to the surrounding media and oxidation. This also explains the stronger background noise for the reference sample in the area of 800.0 to 450.0 eV. A larger part of TiO_2 is exposed during the XPS measurement, causing more electrons from the O 1s of deeper substrate layers to undergo collisions. The peaks for aluminum and especially vanadium are too small to allow a qualitative analysis, though it can be expected that both metals also underwent an oxidation and are present in form of Al_2O_3 and V_2O_3. The values for Ti, Al, V and N are listed in Table 1. The ratio of the three metals does not reflect the nominal elemental composition of Ti64 but this deviation was already reported before.[7] Furthermore, XPS only measures the surface of the substrate with an average depth of approximately 5 nm which is stronger affected by effects like oxidation or adsorption and thus can lead to deviations from the bulk properties. Lastly, the peak at 400.3 eV is the characteristic region for N 1s which shows higher values for all laser-structured samples which again correlates to the higher percentages for C-N at 286.5 eV. Other works also attribute this nitrogen to TiN impurities[8] but this would result in a third peak at around 454.0 eV which is not present for the measured Ti64 samples.

Table 1. Detailed surface composition (at.-%) for Ti 2p, Al 2p, V $2p_{3/2}$ and N 1s.

	Ti 2p	Al 2p	V $2p_{3/2}$	N 1s
Energy [eV]	458.7	74.4	515.8	400.3
Groups	Ti^{4+}	Al^{3+}	V^{3+}	N-C
Reference	13.3	3.6	0.4	0.7
2 µm (air)	2.7	1.2	0.0	7.3
5 µm (air)	3.9	2.1	0.1	4.4
2 µm (water)	2.9	1.2	0.1	8.3
5 µm (water)	5.9	2.4	0.1	5.6
Chemical compounds	TiO_2	Al_2O_3	V_2O_3	contaminations

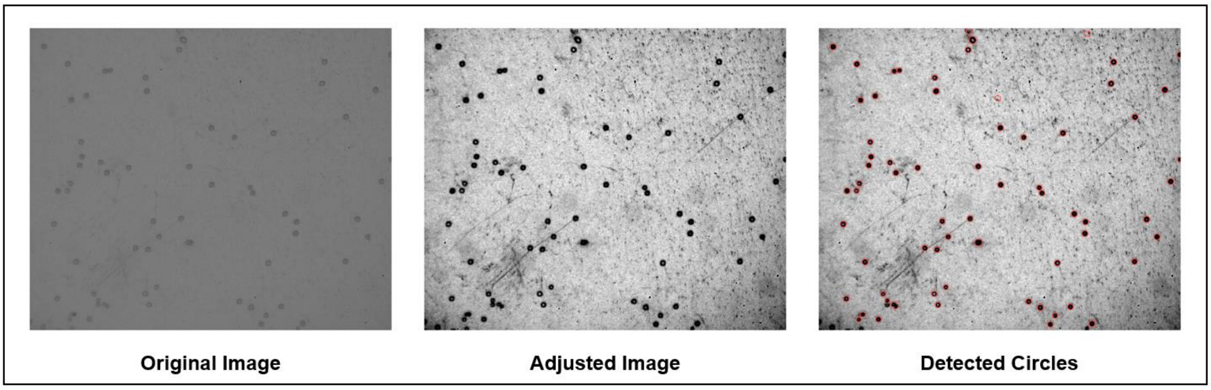

Figure 11. Image processing steps for reference sample at t = 90 s.

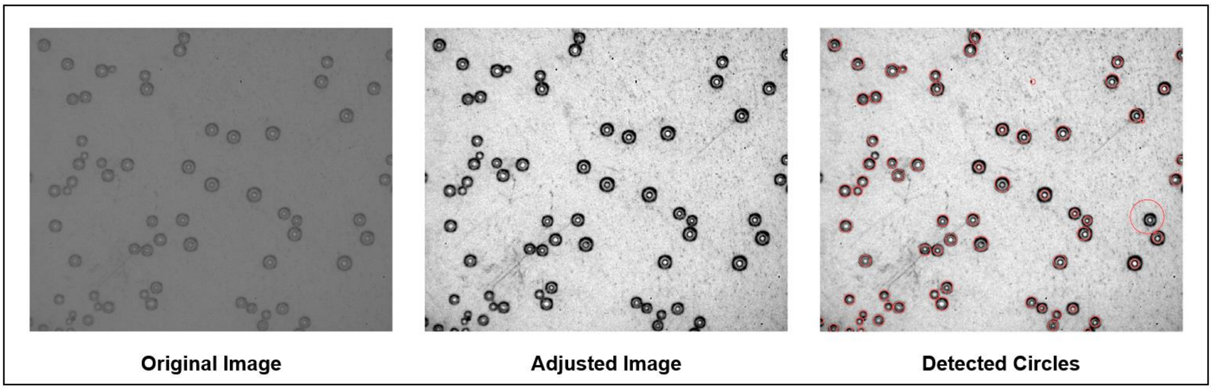

Figure 12. Image processing steps for reference sample at t = 900 s.

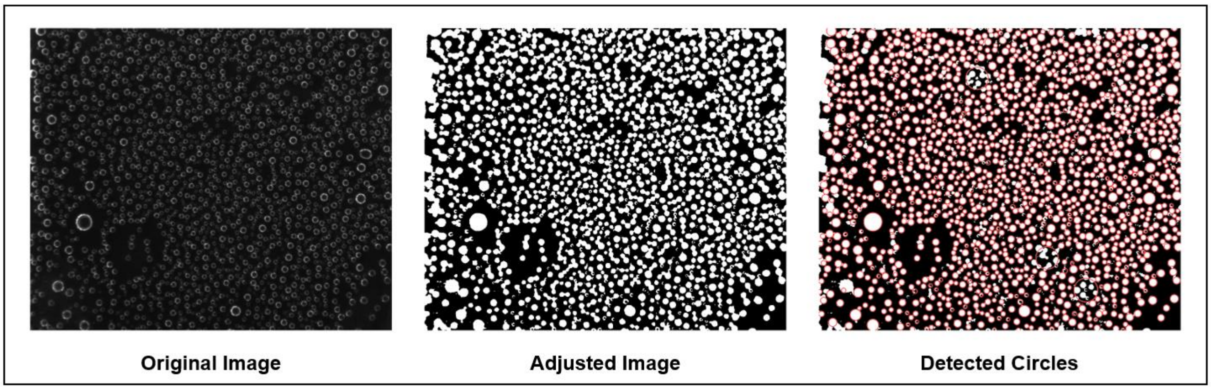

Figure 13. Image processing steps for 5 μm (air) at t = 90 s.

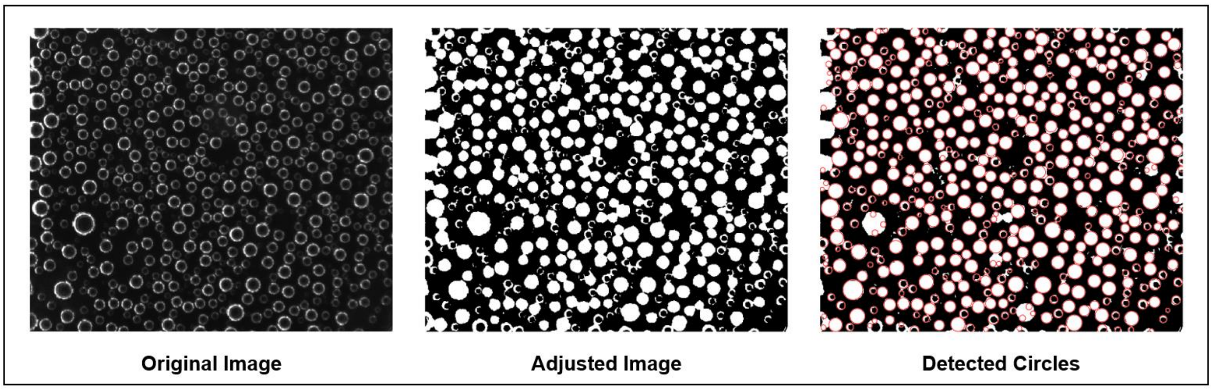

Figure 14. Image processing steps for 5 μm (air) at t = 900 s.

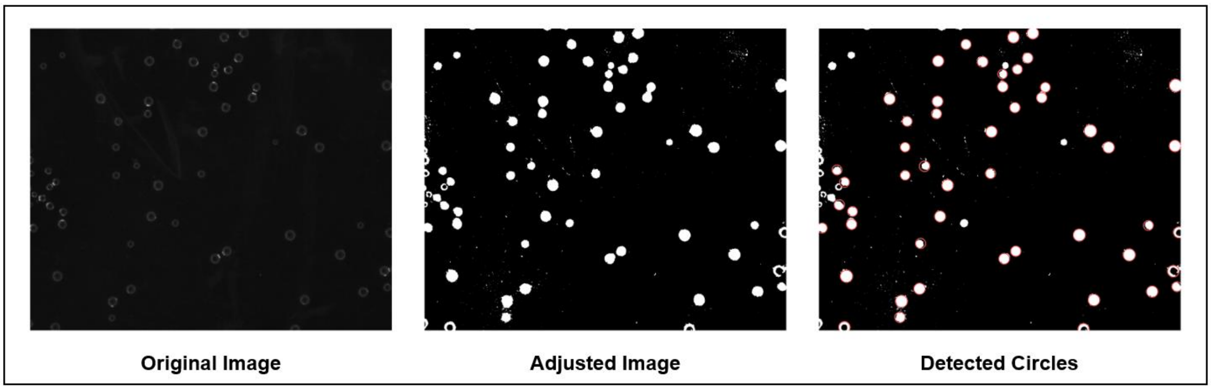

Figure 15. Image processing steps for 5 μm (water) at t = 90 s.

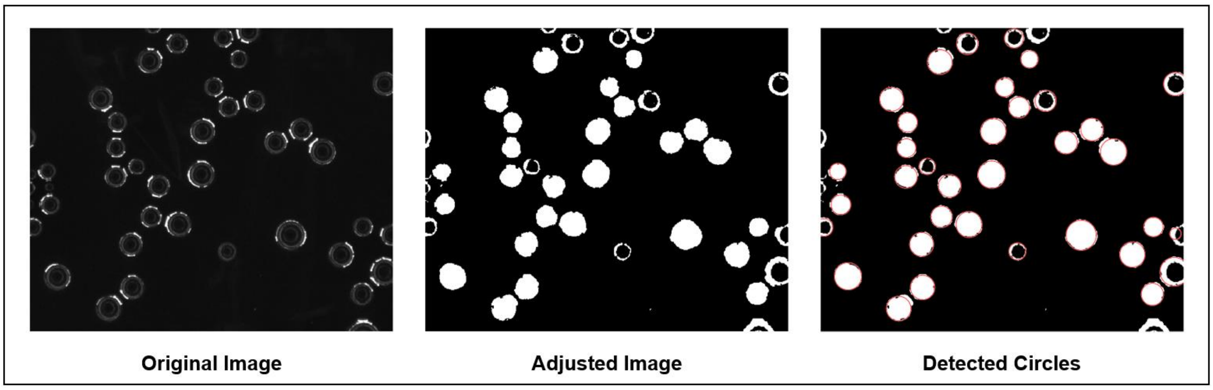

Figure 16. Image processing steps for 5 μm (water) at t = 900 s.

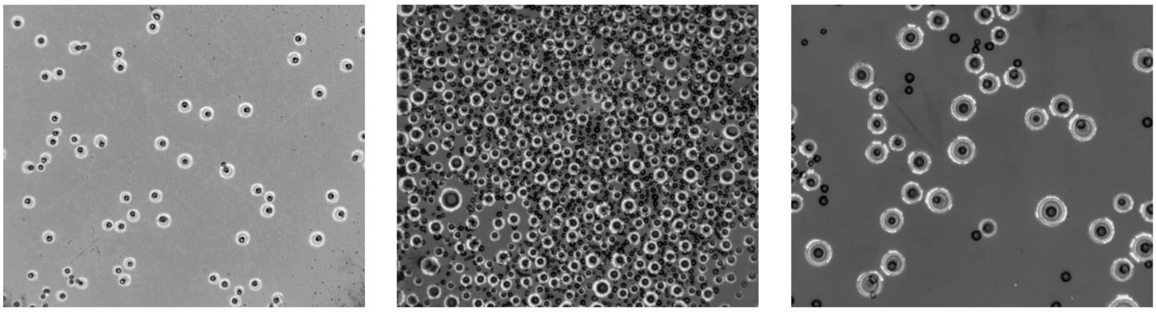

Figure 17. Bubble growth images for reference sample, 5 µm (air) and 5 µm (water). Shown is the visual overlay of the bubbles at t = 90 s (black) und t = 900 s (white) to highlight the differences in size and position during the recording period. The growth can be validated by comparison of the black and white colored bubbles. Black bubbles which do no feature a white counterpart were lost during the recording either due to coalescence with another neighboring bubble or due to detachment. Especially in the reference sample it can be seen that all black bubbles feature a white counterpart or are in its close proximity, indicating that only coalescence and no detachment occurred. In contrast, for the 5 µm (water) sample many standalone black bubbles are noticeable which points towards a higher detachment rate.

Appendix C: O2 Nucleation Videos (avi)

The avi files show the unedited recordings of the O_2 nucleation on the reference, the 5 µm (air) and the 5 µm (water) sample with three recordings for each sample type. The recordings were originally done with 0.1 fps and the generated grey-scale TIFF images were converted into avi format using the Fiji software. Additionally, time stamps were added to the recordings, featuring the time from 90 s to 900 s with an interval of 10 s.

References

(1) Cussler, E. L. *Diffusion: Mass Transfer in Fluid Systems*; Cambridge University Press, **1997**.

(2) Xing, W.; Yin, M.; Lv, Q.; Hu, Y.; Liu, C.; Zhang, J. 1 - Oxygen Solubility, Diffusion Coefficient, and Solution Viscosity. In *Rotating Electrode Methods and Oxygen Reduction Electrocatalysts*; Xing, W., Yin, G., Zhang, J., Eds.; Elsevier: Amsterdam, **2014**; pp 1–31. https://doi.org/10.1016/B978-0-444-63278-4.00001-X.

(3) High Resolution XPS of Organic Polymers: The Scienta ESCA300 Database (Beamson, G.; Briggs, D.). *J. Chem. Educ.* **1993**, *70* (1), A25. https://doi.org/10.1021/ed070pA25.5.

(4) Powell, C. X-Ray Photoelectron Spectroscopy Database XPS,Version 4.1, NIST Standard Reference Database 20, **1989**. https://doi.org/10.18434/T4T88K.

(5) Biesinger, M. C.; Lau, L. W. M.; Gerson, A. R.; Smart, R. St. C. Resolving Surface Chemical States in XPS Analysis of First Row Transition Metals, Oxides and Hydroxides: Sc, Ti, V, Cu and Zn. *Applied Surface Science* **2010**, *257* (3), 887–898. https://doi.org/10.1016/j.apsusc.2010.07.086.

(6) Saha, N. C.; Tompkins, H. G. Titanium Nitride Oxidation Chemistry: An X-ray Photoelectron Spectroscopy Study. *Journal of Applied Physics* **1992**, *72* (7), 3072–3079. https://doi.org/10.1063/1.351465.

(7) Kurtovic, A.; Brandl, E.; Mertens, T.; Maier, H. J. Laser Induced Surface Nano-Structuring of Ti–6Al–4V for Adhesive Bonding. *International Journal of Adhesion and Adhesives* **2013**, *45*, 112–117. https://doi.org/10.1016/j.ijadhadh.2013.05.004.

(8) Bereznai, M.; Pelsöczi, I.; Tóth, Z.; Turzó, K.; Radnai, M.; Bor, Z.; Fazekas, A. Surface Modifications Induced by Ns and Sub-Ps Excimer Laser Pulses on Titanium Implant Material. *Biomaterials* **2003**, *24* (23), 4197–4203. https://doi.org/10.1016/S0142-9612(03)00318-1.
